# An Method of Assessing Lithium-Ion Battery State of Health Based on Interval Belief Rule Base

**DOI:** 10.3390/s26216950

**Published:** 2026-11-03

**Authors:** Hongming Zheng, Yuanyuan Qu, Guangling Zhang

**Affiliations:** 1 School of Computer Science and Information Engineering, Harbin Normal University, Harbin 150025, China; 2 School of Information Engineering, Heilongjiang Agricultural Engineering Vocational and Technical University, Harbin 150080, China

**Keywords:** interval belief rule base, uncertainty quantification, multi-objective composite fitness function, parameter optimization, battery management system, health state assessment, interval evidential reasoning

## Abstract

The accurate assessment of lithium-ion battery State of Health (SOH) is fundamental to the safe and reliable operation of battery management systems. The Belief Rule Base (BRB) integrates quantitative monitoring data and qualitative expert knowledge, thereby supporting SOH assessment. However, existing BRB models produce point-value outputs. The uncertainty of the assessment results cannot be quantified, nor can the belief level of the assessment be characterized. Moreover, information loss is caused by traditional evidential reasoning (ER) when interval belief degrees are processed. Interval reliability is not adequately addressed in existing optimization methods, as interval quality is not incorporated into the objective function. To address these problems, an interval SOH assessment method based on the Interval Belief Rule Base with Interval Evidential Reasoning (IBRB-IER) is proposed. Rule belief degrees are extended from point values to interval forms, enabling uncertainty quantification. Interval information is preserved throughout the fusion process by adopting interval evidential reasoning algorithms. A multi-objective composite fitness function is designed. Global parameter optimization is achieved through the projected covariance matrix adaptation evolution strategy (P-CMA-ES). SOH estimation intervals are finally produced as outputs. The effectiveness of the proposed method in SOH interval assessment is validated through experiments on the NASA dataset.

## 1. Introduction

The accelerating penetration of renewable energy sources into modern power systems has positioned energy storage systems (ESS) as a cornerstone of grid stability, load leveling, and reliable power delivery. Among various ESS technologies, lithium-ion batteries are known for their lightweight design and high energy density. They serve as energy storage components in aerospace vehicles, aircraft, electric vehicles, and portable electronics [1,2]. This broad deployment, however, imposes demanding operational requirements on lithium-ion batteries—high energy density, long cycle life, and intrinsic safety—that ultimately depend on the stability of the electrode materials and the electrochemical interfaces within the cell.

At the material level, achieving these requirements remains a fundamental challenge. For instance, silicon-based anodes, which offer high theoretical capacity for next-generation lithium-ion batteries, suffer from severe structural degradation during cycling due to large volume expansion, and recent studies have attempted to mitigate such degradation through composite design and protective coating strategies [3]. Beyond material-level innovations, the broader issue of battery aging remains a critical concern. With increased service time, internal electrochemical reactions continuously occur, leading to capacity degradation. Gradual performance deterioration is consequently observed. If lithium-ion batteries in an unstable state are continuously used, explosions may be triggered, causing equipment damage, economic losses, or casualties. To avoid such potential disasters caused by battery degradation, system reliability and safety must be ensured. Accurate SOH assessment of lithium-ion batteries is therefore essential [4].

Extensive research efforts have been devoted to the SOH assessment of lithium-ion batteries by both domestic and international scholars. Existing methods are generally classified into three main categories: model-based approaches, data-driven approaches, and hybrid approaches.

Model-based methods fall into three main categories: electrochemical models, equivalent circuit models, and empirical degradation models. Electrochemical models are constructed based on the internal physical and chemical reaction mechanisms of batteries. The battery aging process can be described with relatively high accuracy by these models. However, numerous parameters are involved, identification is difficult, and high computational complexity is exhibited. An electrochemical-based degradation assessment model for the whole life cycle of lithium batteries was developed by SU et al. based on the classical DFN electrochemical model, and accurate reconstruction of internal aging evolution laws was achieved [5]. To address the high computational cost of traditional electrochemical models, a simplified electrochemical model optimized by ensemble learning was proposed by ZHU et al. [6]. Equivalent circuit models are widely used in Battery Management Systems (BMSs) due to their simple structure and limited number of parameters, but their accuracy is limited by model order and parameter identification, making it difficult to fully reflect complex battery degradation behavior. HU et al. constructed a temperature-coupled second-order R-CPE equivalent circuit model, introducing temperature-sensitive parameters to correct polarization characteristics, thereby improving estimation accuracy under varying temperature conditions [7]. However, once a model is established, its expression space is fixed, making it difficult to adaptively capture complex degradation patterns hidden in data. When battery aging behavior deviates from model assumptions, bias correction cannot be achieved through autonomous learning, and inevitable degradation of assessment accuracy is thus caused.

Data-driven methods have attracted extensive attention in recent years, owing to their powerful nonlinear fitting capability and the absence of requirements for accurate physical models. Machine learning methods such as support vector regression, relevance vector machine, and Gaussian process regression have been successfully applied to SOH estimation. A Gaussian process regression model was adopted by LI et al., in which the incremental capacity features of batteries served as inputs, and quantitative assessment of uncertainty caused by noise was achieved. Significant advantages in small-sample assessment were demonstrated [8]. Deep learning methods, such as convolutional neural networks and long short-term memory networks, have further enhanced fitting accuracy. A hybrid CNN-VLSTM deep learning model was constructed by BAO et al., combining convolutional feature extraction with temporal memory to precisely capture long- and short-term dependencies in battery degradation [9]. Nevertheless, data-driven methods generally suffer from poor interpretability; the models are often treated as “black boxes,” and the basis of decision-making is difficult to understand. Furthermore, high requirements for the quantity and quality of training data are imposed by deep models. Overfitting is easily caused under small-sample conditions, and complicated parameter tuning processes are also involved.

To address the respective limitations of model-based and data-driven methods, hybrid methods provide a balanced modeling approach by combining qualitative expert knowledge with quantitative monitoring data, while taking both physical constraints and data fitting accuracy into account. Kalman filtering and its variants achieve state estimation by fusing battery models with measurement data, and satisfactory performance has been demonstrated in joint State of Charge (SOC) and SOH estimation [10]. Reddy et al. developed a hybrid SOH estimation framework integrating fuzzy logic and artificial neural networks, in which a fuzzy inference system provides interpretable baseline estimates through membership functions, and the neural network performs adaptive learning-based correction. By adopting an S-shaped degradation curve to more realistically reflect battery aging behavior, the model achieves more accurate SOH estimation under dynamic operating conditions [11]. An adaptive particle filter model was constructed by YANG et al., in which particle resampling strategies were optimized, alleviating particle degradation and improving battery life prediction accuracy. Among rule-based reasoning methods, fuzzy logic systems handle input–output fuzziness through membership functions [12]. Mbasso et al. developed an interpretable hybrid ANFIS–metaheuristic framework that combines a fuzzy inference system with metaheuristic algorithms such as gray wolf optimization and particle swarm optimization. The fuzzy inference system provides interpretable baseline estimates, while the metaheuristic optimization optimizes membership function parameters and enhances generalization and convergence stability under dynamic conditions [13]. As demonstrated by Wang et al. a physics-guided two-stage residual learning framework can significantly reduce model uncertainty by embedding physical laws into the learning process, thereby improving cross-domain generalization across different vehicles. This physical-guided philosophy is highly complementary to the uncertainty quantification approaches [14]. Other methods include fuzzy logic systems (FLS) and BRB [15,16,17].

The BRB is a novel intelligent expert system proposed by Yang et al., based on Dempster-Shafer evidence theory and fuzzy set theory. Knowledge is represented in the form of IF-THEN rules, in which the conclusion of each rule is expressed as a belief distribution, assigning different degrees of support to different evaluation results. Uncertainties including fuzziness, randomness, and ignorance are effectively handled. The BRB possesses both the interpretability of expert systems and the adaptability of data-driven models: qualitative expert knowledge is integrated, while quantitative information is extracted from data through parameter learning [18,19,20]. In terms of inference mechanism, the ER algorithm is adopted for rule activation and evidence synthesis, thereby providing a solid theoretical foundation and maintaining a transparent inference process [,^21^].

The interval belief rule base (IBRB) is developed based on the traditional BRB by introducing interval forms, thereby significantly enhancing its capability for uncertainty representation and processing [22,23]. In recent years, this method has been applied to lithium battery SOH assessment in several studies [24,25,26]. An IBRB-based SOH estimation method considering expert knowledge credibility (IBRB-c) was proposed by Yin et al., in which fuzzy interpretable intervals were designed as interpretability evaluation criteria [25]. Han et al. proposed a BRB-I model for lithium battery SOH assessment, where structural interpretability and optimization interpretability were constructed through mechanism analysis of lithium batteries, and the whale optimization algorithm (WOA) was adopted for parameter optimization [27]. In addition, Cheng et al. developed an interval-constructed belief rule base with rule reliability (IBRB-r), in which rule combinations were performed in an interval addition manner, thereby avoiding the combinatorial explosion problem caused by the Cartesian product form [28].

Despite these advantages, however, several challenges are faced when using IBRB in lithium battery SOH assessment. First, precise point values are commonly produced as inference outputs by existing BRB models, compressing assessment conclusions into single numerical values [29,30]. As a result, the confidence level of the estimated value cannot be effectively characterized, nor can its reliability be determined. Second, in the few studies that provide interval outputs, fixed-width intervals are generally adopted, so that individual sample differences are ignored. A dilemma then arises in determining the interval width: decision precision is lost when the width is too wide, whereas coverage probability is reduced when the width is too narrow. Therefore, a reasonable balance between interval width and coverage probability must be established, and a mechanism for adaptively generating compact and reliable prediction intervals needs to be designed—a gap that remains to be addressed. Third, a collaborative optimization mechanism for interval coverage and width is currently lacking. Point estimation accuracy is commonly used as the sole objective in parameter optimization; even when interval coverage probability (ICP) is employed as an evaluation metric in some studies, it is only applied in the post-evaluation stage and is not incorporated into the fitness function design. Furthermore, from a practical engineering perspective, it must be acknowledged that battery state parameters are often highly coupled in real-world BMS applications. As highlighted by recent research, the precise joint estimation of SOC and temperature is critical for battery state assessment, and advanced techniques like ultrasonic reflection can decouple these parameters under complex conditions [31]. Since temperature significantly affects electrochemical aging mechanisms (e.g., accelerating SEI growth at high temperatures), different operating conditions inherently induce varying degrees of uncertainty in the data. While the primary focus of this study is SOH estimation, addressing this multi-parameter coupling and improving robustness under varying temperatures remains a crucial future direction.

To address the above issues, an interval SOH assessment method based on IBRB-IER is proposed. In this method, the belief degrees of rules are extended from precise point values to belief degree intervals, thereby enabling uncertainty quantification in the assessment process. An interval evidential reasoning (IER) algorithm is adopted to maintain complete propagation of interval information during rule fusion, so that SOH estimation intervals are eventually produced as outputs. In addition, a multi-objective composite fitness function integrating mean squared error, coverage loss, and width loss is designed, and global parameter optimization is achieved through the P-CMA-ES algorithm with a projection operator [32,33]. As a result, the assessment performance is significantly improved, and the true health state of the system is more accurately reflected by the IBRB-IER model.

To clearly highlight the theoretical innovations of the proposed method, a systematic comparison between the IBRB-IER model and existing IBRB variants (i.e., IBRB-c, BRB-I, and IBRB-r) is summarized in Table 1. The comparison is conducted from three perspectives: uncertainty representation, interval inference mechanism, and optimization objective function. As shown in the table, existing methods either rely on point outputs or suffer from information loss during interval fusion. The IBRB-IER model fundamentally addresses these limitations by introducing interval belief degrees, maintaining upper-lower bound propagation, and designing a multi-objective fitness function.

The remainder of this paper is organized as follows. In Section 1, the current research status of lithium-ion battery SOH assessment is reviewed, and the IBRB-IER model is proposed. Section 2 discusses the main challenges addressed during model construction. Section 3 systematically presents the proposed model from four perspectives: model construction, rule activation, inference, and optimization. Section 4 verifies the model effectiveness through experimental and comparative analyses on lithium-ion battery data. Section 5 summarizes the key findings and outlines potential directions for future research.

## 2. Problem Description

The construction of a health state assessment model for lithium-ion batteries based on IBRB-IER needs to address the following three problems.

Problem 1: How can the uncertainty in the SOH assessment process be fully quantified?

Traditional SOH assessment methods commonly output single deterministic numerical values, making it difficult to characterize the credibility and risk level of the assessment results. To address this limitation, a modeling framework based on the IBRB is introduced, in which the output belief degree of each rule is extended from point values to belief degree intervals. Through the IER algorithm, systematic propagation and accumulation of uncertainty are realized, and SOH prediction intervals are thereby produced as outputs.

(1) $$[[\beta_{n}^{L},\beta_{n}^{U}]=\Phi \left({\left\{w_{j},r_{j},[\beta_{j,n}^{L},\beta_{j,n}^{U}]\right\}}_{j=1}^{M}\right)$$

where $\left[\beta_{j,n}^{L},\beta_{j,n}^{U}\right]$ denotes the belief degree interval of the nth evaluation grade in the jth rule. w_j_ and r_j_ represent the rule weight and the reliability factor, respectively. Φ(·) is defined as the recursive combination operator of interval evidential reasoning. M is the total number of activated rules. N is the number of evaluation grades. The final output belief degree interval $\left[\beta_{n}^{L},\beta_{n}^{U}\right]$ is generated. The uncertainty degree of the SOH assessment results is directly quantified by this interval.

Problem 2: How can reasonable SOH prediction intervals be constructed?

Fixed-width strategies are commonly adopted in existing interval construction methods, thereby ignoring individual sample differences and failing to reflect variations in uncertainty degrees under different operating conditions. Moreover, a dilemma arises in determining the interval width: decision precision is lost when the width is too wide, whereas coverage probability is reduced when the width is too narrow. To address this issue, the final SOH prediction intervals are derived from the interval belief distribution through a utility function transformation within the interval evidential reasoning framework, as follows:

(2) $$[{\mathrm{SOH}}_{\mathrm{Low}},{\mathrm{SOH}}_{\mathrm{Up}}]=T\left([\beta_{n}^{L},\beta_{n}^{U}],u_{n}\right)$$

where $\left[\beta_{n}^{L},\beta_{n}^{U}\right]$ denotes the belief degree interval of the nth evaluation grade, which is output by interval evidential reasoning. u_n_ represents the utility value corresponding to the ith evaluation grade. T(·) is defined as the utility transformation function from the interval belief distribution to the numerical interval. The final prediction interval is obtained as [SOH_Low_,SOH_Up_].

Problem 3: How can collaborative optimization of interval coverage and width be achieved?

Point estimation accuracy is commonly used as the sole optimization objective in existing parameter optimization methods, while interval quality metrics are not incorporated into the fitness function design. As a consequence, a trade-off arises in the optimized models: either the intervals are too wide with high coverage, or they are compact with insufficient coverage. To address this issue, a multi-objective composite fitness function is designed in this paper, integrating point estimation accuracy with interval quality metrics as follows:

(3) $$\min_{\theta }F(\theta )=F_{\mathrm{MSE}}(\theta )+\lambda_{\mathrm{cov}}\cdot F_{\mathrm{cov}}(\theta )+\lambda_{\mathrm{width}}\cdot F_{\mathrm{width}}(\theta )+P(\theta )$$

where θ denotes the set of model parameters to be optimized. ℱ_MSE_(θ) represents the mean squared error of point estimates. ℱ_cov_(θ) is the penalty term for interval coverage probability. ℱ_width_(θ) is the penalty term for interval width. λ_cov_ and λ_width_ are the corresponding balance coefficients.

## 3. Construction of IBRB-IER

**Figure 1:**
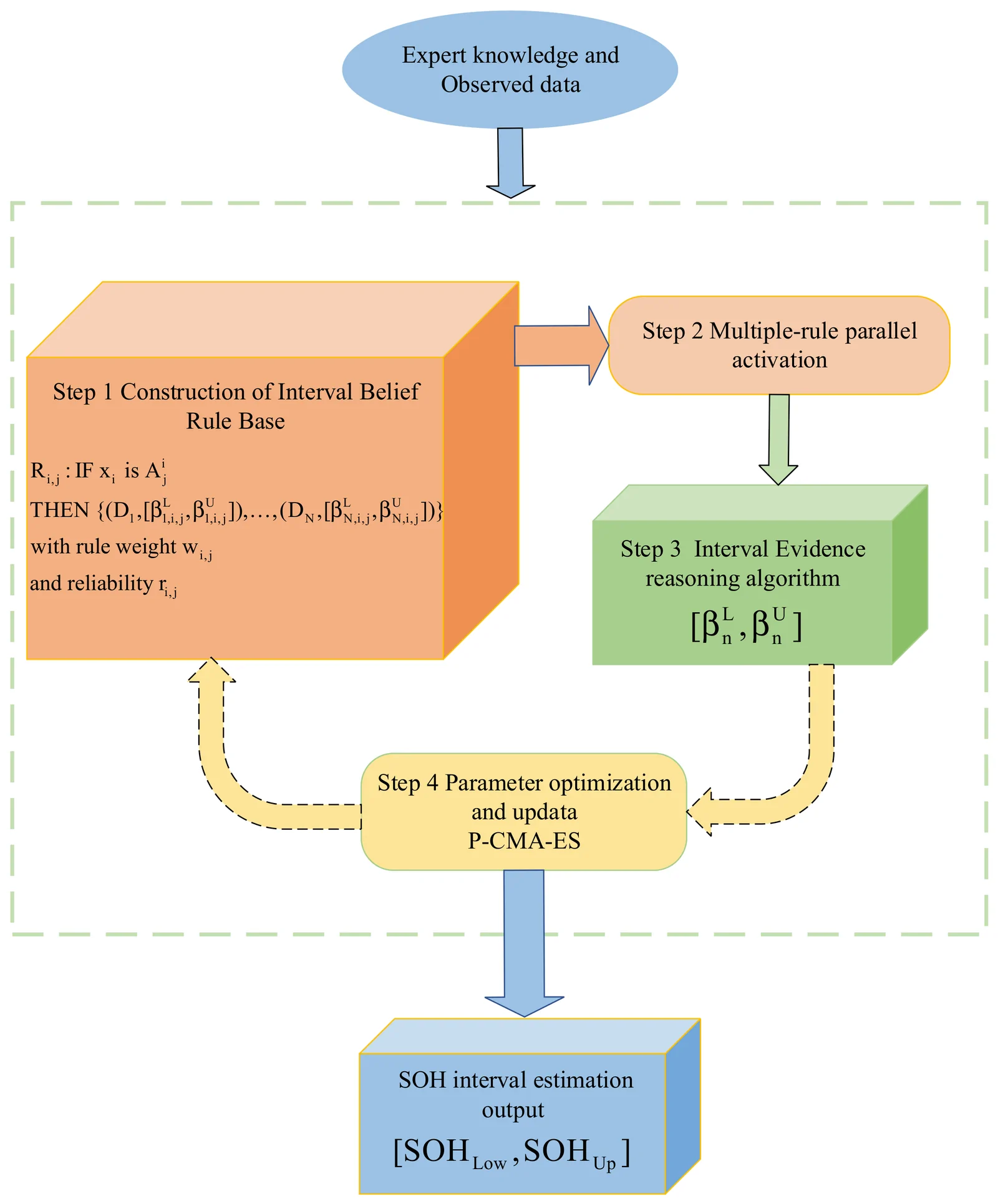
Overall framework of the IBRB-IER model.

To address the above issues, an interval SOH assessment method based on IBRB-IER is proposed in this study. The model is constructed through the organic integration of four components: interval rule construction, parallel activation of multiple rules, interval evidential reasoning fusion, and P-CMA-ES parameter optimization. Battery aging monitoring data and expert knowledge derived from degradation laws are employed as model inputs. Attribute matching degrees are first calculated using Gaussian membership functions, while rule uncertainties are characterized by interval belief degrees. The interval evidential reasoning algorithm then performs fusion of the activated rules, and SOH estimation intervals are finally produced as outputs. Meanwhile, global optimization of rule parameters is conducted using P-CMA-ES, with a comprehensive fitness function composed of mean squared error, coverage loss, and width loss being minimized. In this way, both accuracy and compactness of the interval estimates are ensured. The overall framework is illustrated in Figure 1.

### 3.1. Construction of the IBRB-IER Model

#### 3.1.1. Interval Rule Construction

In this paper, the rule base is constructed in an interval manner. The core idea is stated as follows: each attribute possesses its own set of rules independently. It is not necessary to construct rules for all possible combinations of all attributes.

There are M antecedent attributes. For the ith antecedent attribute x_i_(i = 1,2,...,M), J_i_ reference intervals are defined, denoted as $A_{i}=\{A_{1}^{i},A_{2}^{i},\ldots ,A_{J_{i}}^{i}\}$. The interval belief rule table for the ith attribute is defined as follows:

(4) $$R_{i}=\{R_{i,j}:j=1,2,\ldots ,J_{i}\}$$

The kth rule $R_{i,j}$ is formulated as follows:

(5) $$\begin{array}{l} R_{i,j}:\mathrm{IF}\;x_{i}\;\mathrm{is}\;A_{j}^{i}\\ \mathrm{THEN}\;\{(D_{1},[\beta_{1,i,j}^{L},\beta_{1,i,j}^{U}]),\ldots ,(D_{N},[\beta_{N,i,j}^{L},\beta_{N,i,j}^{U}])\}\\ \mathrm{with}\;\mathrm{rule}\;\mathrm{weight}\;w_{i,j}\;\\ \mathrm{and}\;\mathrm{reliability}\;r_{i,j} \end{array}$$

where D_n_ denotes the nth evaluation grade, where (n = 1,2,…,N). The term $\left[\beta_{n,i,j}^{L},\beta_{n,i,j}^{U}\right]$ represents the belief degree interval assigned to outcome D_n_ in the jth rule of the ith attribute. The following constraints are satisfied:

(6) $$0\leq \beta_{n,i,j}^{L}\leq \beta_{n,i,j}^{U}\leq 1$$

(7) $$\sum_{n=1}^{N}\beta_{n,i,j}^{L}\leq 1\leq \sum_{n=1}^{N}\beta_{n,i,j}^{U}$$

where the true belief distribution lies within the interval $\left[\beta_{n,i,j}^{L},\beta_{n,i,j}^{U}\right]$, but its exact value is unknown. The uncertainty of each rule’s output is quantified through this interval representation.

#### 3.1.2. Parameterization of Rule Belief Degree Intervals

To facilitate parameter optimization, the belief degree interval of each rule is parameterized by the midpoint p_n,i,j_ and the half-width δ_i,j_:

(8) $$\beta_{n,i,j}^{L}=\max(0,p_{n,i,j}-\delta_{i,j})$$

(9) $$\beta_{n,i,j}^{U}=\min(1,p_{n,i,j}+\delta_{i,j})$$

where p_n,i,j_ ∈ [0,1] represents the midpoint of the belief degree assigned to outcome D_n_ in the jth rule of the ith attribute. The following normalization constraint is satisfied:

(10) $$\sum_{n=1}^{N}p_{n,i,j}=1$$

where δ_i,j_ ∈ [0,0.5] denotes the interval half-width in the jth rule of the ith attribute. The uncertainty degree of the rule output is controlled by this parameter. A zero value of δ_i,j_ = 0 indicates that a precise belief distribution is output by the rule. A larger value of δ_i,j_ implies a higher level of uncertainty.

#### 3.1.3. Rule Reliability and Weight

In this paper, a reliability parameter r_i,j_ and a rule weight w_i,j_ are introduced for each rule.

The reliability r_i,j_ ∈ [0,1] of the jth rule $R_{i,j}$ in the ith attribute represents the credibility of its output. A higher reliability leads to a larger contribution of this rule during evidence fusion. A value of r_i,j_ = 1 indicates that the rule is fully reliable. A value of r_i,j_ = 0 indicates that the rule is completely unreliable.

The weight w_i,j_ ∈ [0,1] of the jth rule $R_{i,j}$ in the ith attribute represents its relative importance within the rule base. Prior expert judgments on the importance of different rules are reflected by the rule weights.

In summary, the parameter set to be optimized in the IBRB-IER model is given as follows:

(11) Θ = {p_n,i,j_,δ_i,j_,r_i,j_,w_i,j_∣i = 1,…,M;j = 1,…,J_i_;n = 1,…,N}

For each rule of each attribute, N nominal belief degree parameters p_n,i,j_, one interval half-width δ_i,j_, one reliability parameter r_i,j_, and one rule weight w_i,j_ are included.

### 3.2. Rule Activation Mechanism of IBRB-IER

#### 3.2.1. Gaussian Membership Function

For each input sample x = (x_1_,x_2_,…,x_M_), the matching degree between each attribute value and each reference level needs to be calculated. A Gaussian membership function is adopted in this paper to smoothly characterize the matching relationship between attribute values and reference levels.

The Gaussian membership function corresponding to the jth reference level $A_{j}^{i}$ of the ith attribute is defined as follows:

(12) $$\mu_{i,j}(x_{i})=\exp\left(-\frac{1}{2}{\left(\frac{x_{i}-c_{i,j}}{\sigma_{i,j}}\right)}^{2}\right),\;i=1,2,\ldots ,M,j=1,2,\ldots ,J_{i}$$

where c_i,j_ denotes the center value (midpoint of the interval) of the jth reference level for the ith attribute, and σ_i,j_ is the width parameter.

The center value of each reference level is determined by its neighboring reference values. The reference value sequence of the ith attribute is given as $V_{i}=\{v_{i}^{0},v_{i}^{1},\ldots ,v_{i}^{J_{i}}\}$. Where $v_{i}^{j-1}$ and $v_{i}^{j}$ represent the lower and upper bounds of the jth interval, respectively. The center value is then computed as:

(13) $$c_{i,j}=\frac{v_{i}^{j-1}+v_{i}^{j}}{2}$$

The width parameter is set as γ.

(14) $$\sigma_{i,j}=\gamma \cdot (v_{i}^{j}-v_{i}^{j-1})$$

where γ is a scaling factor.

#### 3.2.2. Normalization of Membership Degrees

To avoid the influence of dimensional differences in membership degrees across different attributes, the membership degrees of each attribute are normalized as follows:

(15) $${\tilde{\mu }}_{i,j}(x_{i})=\frac{\mu_{i,j}(x_{i})}{\sum_{k=1}^{J_{i}}\mu_{i,k}(x_{i})}$$

The normalized membership degrees satisfy the following constraints:

(16) $$\sum_{j=1}^{J_{i}}{\tilde{\mu }}_{i,j}(x_{i})=1$$

#### 3.2.3. Rule Matching Degree

For an input sample x, the matching degree of the jth rule $R_{i,j}$ for the ith attribute is defined as the normalized membership degree:

(17) $$\alpha_{i,j}(x)={\tilde{\mu }}_{i,j}(x_{i})$$

The matching degree α_i,j_(x) ∈ [0,1] reflects the relevance between the current input and the $R_{i,j}$ rule. A larger value indicates greater relevance of this rule to the current input.

#### 3.2.4. Parallel Activation of Multiple Rules

An activation threshold τ is set. When α_i,j_(x) > τ, the rule $R_{i,j}$ is activated. The set of all activated rules is termed the activated rule set:

(18) R_act_(x) = {R_i,j_:α_i,j_(x) > τ,i = 1,…,M,j = 1,…,J_i_}

Due to the smoothness property of the Gaussian membership function, each attribute value typically activates 1~3 adjacent reference levels simultaneously. Therefore, the total number of activated rules L_act_ = |ℛ_act_(x)| generally satisfies the following condition:

(19) M ≤ L_act_ ≤ 3M

A special case is handled as follows. If ℛ_act_(x)=∅ (no rule is activated), the rule with the maximum matching degree for each attribute is selected as the activated rule:

(20) $$R_{\mathrm{act}}(x)=\left\{R_{i,j_{i}^{*}}:j_{i}^{*}=\arg\max_{1\leq j\leq J_{i}}\alpha_{i,j}(x),i=1,\ldots ,M\right\}$$

#### 3.2.5. Global Rule Indexing

To facilitate subsequent interval evidential reasoning, the activated rules are mapped to a global index space. A global index mapping function is defined as follows:

(21) $$g(i,j)=\sum_{k=1}^{i-1}J_{k}+j$$

where g(i,j) ∈ {1,2,…,L_IBRB_} denotes the global index of the jth rule in the ith attribute. The corresponding global index set for the activated rule set R_act_ is given as:

(22) J_act_(x) = {g(i,j):R_i,j_ ∈ R_act_(x)}

The corresponding matching degree vector is given as:

(23) $$a_{\mathrm{act}}(x)={\left[a_{i,j}(x):g(i,j)\in J_{\mathrm{act}}(x)\right]}^{T}\in {\mathbb{R}}^{L_{\mathrm{act}}}$$

### 3.3. Inference of the IBRB-IER Model

The IER is an extension of the classical D-S evidence theory. Uncertainty information is converted into interval-valued BPAs, denoted as m:2^Θ^ → {[a,b]∣0 ≤ a ≤ b ≤ 1}. This interval representation offers several theoretical advantages. An interval BPA contains both central tendency and uncertainty range information. For an interval BPA [m^L^,m^U^], the most likely probability mass estimate is provided by (m^L^ + m^U^)/2, while the uncertainty degree of this estimate is quantified by the interval width m^U^ − m^L^. Human judgments of uncertainty are inherently interval-valued: experts typically state that “the confidence is approximately between 0.6 and 0.8,” rather than “the confidence is exactly 0.7.” This cognitive pattern is more naturally reflected by interval BPAs. During recursive combination, the propagation of uncertainty from input to output can be tracked layer by layer through interval BPAs, and the combined interval width directly reflects the aggregated uncertainty from multiple sources.

To illustrate the advantages of interval inference over precise inference, consider the following example. In a battery SOH assessment problem, two rules are activated:

Rule R_1_: IF internal resistance is high, THEN SOH is low, with a belief degree interval of [0.6, 0.8]. Rule R_2_: IF capacity is low, THEN SOH is low, with a belief degree interval of [0.5, 0.7].

Precise inference approach: The two intervals are degenerated into midpoints of 0.7 and 0.6, respectively. After ER combination, a single belief degree value (approximately 0.65) is obtained. However, the uncertainty information contained in the original intervals is lost, and the reliability of this value cannot be judged by decision-makers.

Interval inference approach: The interval forms are preserved. After IER combination, a belief degree interval (approximately [0.55, 0.75]) is obtained. Not only is the central estimate of 0.65 provided, but it is also clearly indicated that the true belief degree is likely to fall within [0.55, 0.75]. Based on this result, a conservative decision can be made using the lower bound of 0.55, while an optimistic decision can be made using the upper bound of 0.75.

The above example demonstrates that significant advantages in preserving information completeness and supporting decision-making are possessed by interval inference.

#### 3.3.1. Activation Rules and Weight Calculation

To facilitate describing the recursive combination process in interval evidential reasoning, each rule in the activated rule set R_act_(x) is treated as an independent piece of evidence. For ease of presentation, the activated rules are re-indexed by their global indices g(i,j) as k = 1,2,…,L_act_. where L_act_ = |ℛ_act_(x)| is the total number of activated rules.

For the kth activated rule R_k_, its interval belief degree vector is denoted as $\left[\beta_{k}^{L},\beta_{k}^{U}]=\{[\beta_{k,1}^{L},\beta_{k,1}^{U}],\ldots ,[\beta_{k,N}^{L},\beta_{k,N}^{U}]\right\}$, the reliability factor is denoted as r_k_, the rule weight is denoted as w_k_, and the matching degree is denoted as α_k_.

The activation weight of the kth activated rule R_k_ is defined as:

(24) $$\omega_{k}=\frac{w_{k}\cdot r_{k}\cdot \alpha_{k}}{\sum_{l=1}^{L_{\mathrm{act}}}w_{l}\cdot r_{l}\cdot \alpha_{l}}$$

where w_k_ is the rule weight, r_k_ is the reliability factor, and α_k_ is the matching degree. The activation weights satisfy the following constraint:

(25) $$\sum_{k=1}^{L_{\mathrm{act}}}\omega_{k}=1,\;\omega_{k}\geq 0$$

#### 3.3.2. Interval Basic Probability Mass

The interval basic probability mass (BPM) of the kth activated rule is defined as follows.

For each evaluation grade D_n_(n = 1,…,N):

(26) $$m_{n,k}^{L}=\omega_{k}\cdot \beta_{k,n}^{L}$$

(27) $$m_{n,k}^{U}=\omega_{k}\cdot \beta_{k,n}^{U}$$

The interval basic probability mass assigned to the whole set D (representing uncertainty) by the kth activated rule is given as:

(28) $$m_{D,k}^{L}=\max\left(0,1-\omega_{k}\cdot \sum_{n=1}^{N}\beta_{k,n}^{U}\right)$$

(29) $$m_{D,k}^{U}=\min\left(1,1-\omega_{k}\cdot \sum_{n=1}^{N}\beta_{k,n}^{L}\right)$$

#### 3.3.3. Recursive Combination of Interval Evidential Reasoning

The core idea of the interval ER algorithm is to strictly maintain the propagation of lower and upper bounds throughout the recursive combination process. Unlike traditional ER, which degenerates intervals into midpoints for single-path inference, interval ER maintains two parallel inference paths simultaneously: a lower-bound path and an upper-bound path.

Assume that the interval probability masses of the first k − 1 pieces of evidence are denoted as $\left(m_{n,\mathrm{comb}}^{L},m_{n,\mathrm{comb}}^{U}\right)$ and $\left(m_{D,\mathrm{comb}}^{L},m_{D,\mathrm{comb}}^{U}\right)$. The interval probability masses of the kth new piece of evidence are denoted as $\left(m_{n,k}^{L},m_{n,k}^{U}\right)$ and $\left(m_{D,k}^{L},m_{D,k}^{U}\right)$. The interval ER combination of the two pieces of evidence is performed.

Step 1: Calculation of the interval conflict coefficient

The conflict coefficient measures the degree of conflict between two pieces of evidence.

(30) $$k_{k}^{L}=(1-m_{D,\mathrm{comb}}^{U})(1-m_{D,k}^{U})-\sum_{n=1}^{N}m_{n,\mathrm{comb}}^{U}m_{n,k}^{U}$$

(31) $$k_{k}^{U}=(1-m_{D,\mathrm{comb}}^{L})(1-m_{D,k}^{L})-\sum_{n=1}^{N}m_{n,\mathrm{comb}}^{L}m_{n,k}^{L}$$

Step 2: Calculation of the K_k_ interval normalization factor

(32) $$K_{k}^{L}=\frac{1}{1-k_{k}^{U}}\;K_{k}^{U}=\frac{1}{1-k_{k}^{L}}$$

To ensure that the denominator is positive, the following treatment is applied. If $1-k_{k}^{U}\leq 0$, then $K_{k}^{L}=1/\varepsilon$ is set to ε, where ε is a small positive number.

Step 3: Combination of probability masses for each evaluation grade

For each evaluation grade n = 1,…,N:

(33) $$m_{n,\mathrm{new}}^{L}=K_{k}^{L}\cdot (m_{n,\mathrm{comb}}^{L}m_{n,k}^{L}+m_{n,\mathrm{comb}}^{L}m_{D,k}^{L}+m_{D,\mathrm{comb}}^{L}m_{n,k}^{L})$$

(34) $$m_{n,\mathrm{new}}^{U}=K_{k}^{U}\cdot (m_{n,\mathrm{comb}}^{U}m_{n,k}^{U}+m_{n,\mathrm{comb}}^{U}m_{D,k}^{U}+m_{D,\mathrm{comb}}^{U}m_{n,k}^{U})$$

where the combined m_n_ is derived from three sources: both pieces of evidence support D_n_: (m_n,comb_m_n,k_); evidence 1 supports D_n_ and evidence 2 is uncertain: (m_n,comb_m_D,k_); evidence 1 is uncertain and evidence 2 supports D_n_: (m_D,comb_m_n,k_).

Step 4: Combination of the probability mass for uncertainty

(35) $$m_{D,\mathrm{new}}^{L}=K_{k}^{L}\cdot m_{D,\mathrm{comb}}^{L}m_{D,k}^{L}$$

(36) $$m_{D,\mathrm{new}}^{U}=K_{k}^{U}\cdot m_{D,\mathrm{comb}}^{U}m_{D,k}^{U}$$

Uncertainty is generated only when both pieces of evidence are uncertain.

Step 5: Recursive iteration

The combined result is updated as follows:

(37) $$m_{n,\mathrm{comb}}^{L}\leftarrow m_{n,\mathrm{new}}^{L},\;m_{n,\mathrm{comb}}^{U}\leftarrow m_{n,\mathrm{new}}^{U},\;m_{D,\mathrm{comb}}^{L}\leftarrow m_{D,\mathrm{new}}^{L},\;m_{D,\mathrm{comb}}^{U}\leftarrow m_{D,\mathrm{new}}^{U}$$

**Figure 2:**
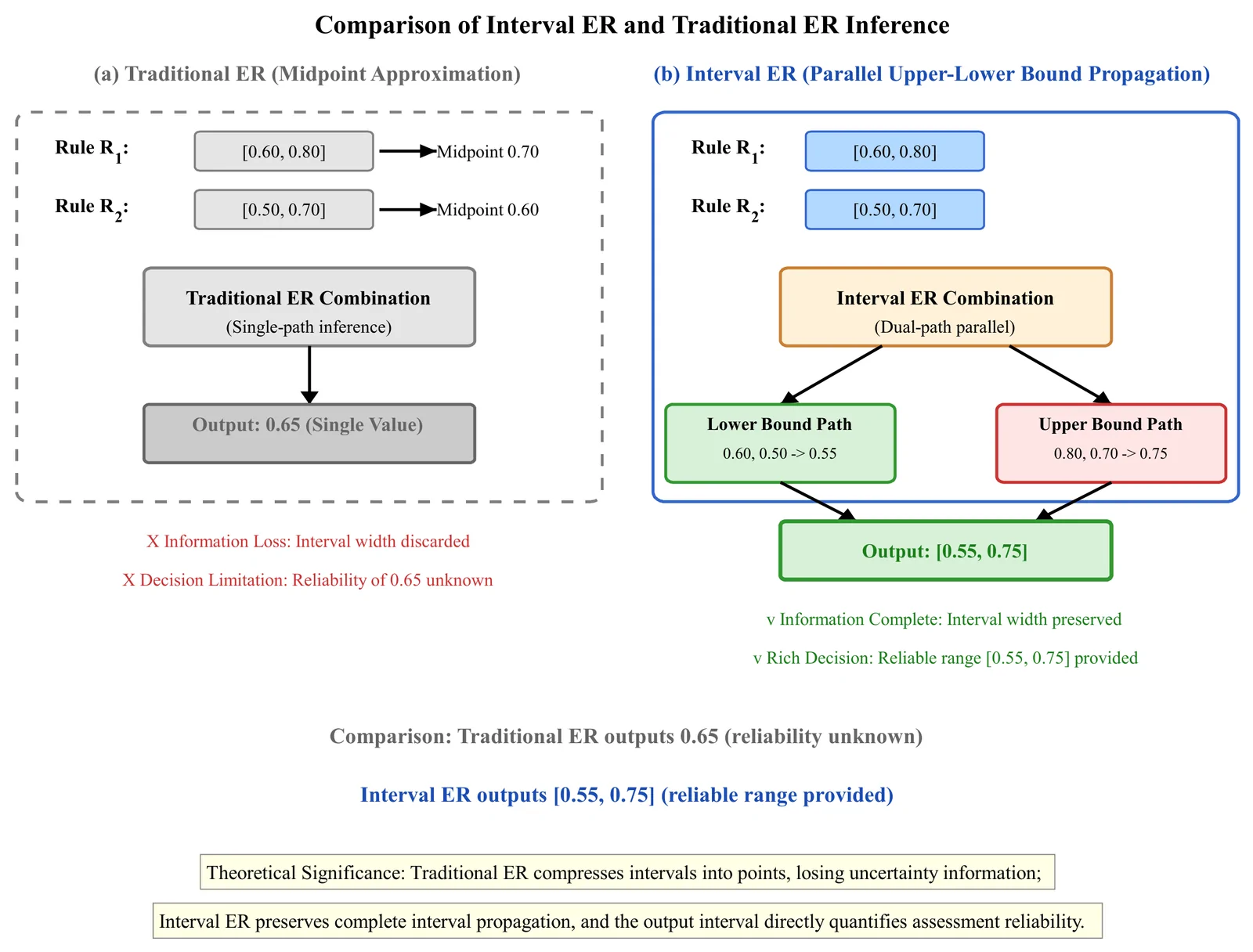
Comparison of Interval ER and Traditional ER Inference.

The above combination steps are performed recursively for all activated rules. The inference processes of interval ER and traditional ER are compared in Figure 2 to illustrate the advantage of interval ER in avoiding information loss.

#### 3.3.4. Calculation of Final Belief Degree Intervals

After all activated rules are recursively combined, the final interval basic probability masses $(m_{n,\mathrm{final}}^{L},m_{n,\mathrm{final}}^{U})\;$ and $\left(m_{D,\mathrm{final}}^{L},m_{D,\mathrm{final}}^{U}\right)$ are obtained. According to evidence theory, the relationship between the final belief degree β_n_ and the final basic probability mass m_n_ is given as:

(38) $$\beta_{n}=\frac{m_{n}}{1-m_{D}}$$

Since both m_n_ and m_D_ are interval-valued, the final belief degree interval is obtained according to interval arithmetic rules as follows:

(39) $$\beta_{n}^{L}=\frac{m_{n,\mathrm{final}}^{L}}{1-m_{D,\mathrm{final}}^{U}}$$

(40) $$\beta_{n}^{U}=\frac{m_{n,\mathrm{final}}^{U}}{1-m_{D,\mathrm{final}}^{L}}$$

The final belief degree intervals $\beta^{L}=[\beta_{1}^{L},\beta_{2}^{L},\ldots ,\beta_{N}^{L}]$ and $\beta^{U}=[\beta_{1}^{U},\beta_{2}^{U},\ldots ,\beta_{N}^{U}]$ satisfy the following constraints:

(41) $$0\leq \beta_{n}^{L}\leq \beta_{n}^{U}\leq 1,\;\sum_{n=1}^{N}\beta_{n}^{L}\leq 1\leq \sum_{n=1}^{N}\beta_{n}^{U}$$

#### 3.3.5. Point Estimation and Interval Estimation of SOH

Let the utility value corresponding to the evaluation grade D_n_ be denoted as u_n_. Then,

SOH point estimate (expected utility):

(42) $$\hat{y}=\sum_{n=1}^{N}\left(\frac{\beta_{n}^{L}+\beta_{n}^{U}}{2}\cdot u_{n}\right)$$

Lower bound of the SOH interval:

(43) $$y_{L}=\sum_{n=1}^{N}\beta_{n}^{L}\cdot u_{n}$$

Upper bound of the SOH interval:

(44) $$y_{U}=\sum_{n=1}^{N}\beta_{n}^{U}\cdot u_{n}$$

The final output is given as $\left[y_{L},\hat{y},y_{U}\right]$, where $\hat{y}$ is the point estimate of SOH, and [y_L_,y_U_] is the estimation interval of SOH.

(45) $$0\leq y_{L}\leq \hat{y}\leq y_{U}\leq 1$$

### 3.4. Optimization of the IBRB-IER Model

The parameter optimization of the IBRB-IER model is formulated as a constrained nonlinear optimization problem. The P-CMA-ES is adopted in this paper for global optimization of the model parameters.

#### 3.4.1. Optimization Variables and Constraints

The parameter vector to be optimized in the IBRB-IER model is given as:

(46) $$\theta ={\left[\begin{array}{c} p_{1,1,1},\ldots ,p_{N,M,J_{M}},\delta_{1,1},\ldots ,\delta_{M,J_{M}},r_{1,1},\ldots ,r_{M,J_{M}},w_{1,1},\ldots ,w_{M,J_{M}} \end{array}\right]}^{T}$$

The optimization variables are required to satisfy the following constraints:

Constraint C1 (Normalization of belief degree midpoints):

(47) $$\sum_{n=1}^{N}p_{n,i,j}=1,\forall i=1,\ldots ,M,\forall j=1,\ldots ,J_{i}$$

Constraint C2 (Variable bounds):

(48) 0 ≤ p_n,i,j_ ≤ 1, 0 ≤ δ_i,j_ ≤ 0.5, 0 ≤ r_i,j_ ≤ 1, 0 ≤ w_i,j_ ≤ 1

#### 3.4.2. Projection Operator for Equality Constraints

Constraint C1 is an equality constraint, formulated as AΘ = **1**, where $A\in {\mathbb{R}}^{L_{\mathrm{IBRB}}\times (L_{\mathrm{IBRB}}\times N)}$ is a sparse matrix. A projection operator is adopted in this paper to handle this constraint.

For any candidate solution Θ, if its belief degree midpoint component p_n,i,j_ does not satisfy the normalization constraint, it is mapped to the feasible region through projection:

(49) $${\tilde{p}}_{n,i,j}=\frac{p_{n,i,j}}{\sum_{k=1}^{N}p_{k,i,j}}$$

After projection, the transformed solution satisfies $\sum_{n=1}^{N}{\tilde{p}}_{n,i,j}=1$ The projection operator is applied to all candidate solutions in each generation of CMA-ES.

#### 3.4.3. Fitness Function Design

The fitness function designed in this paper comprehensively considers three objectives: point estimation accuracy, interval coverage probability, and interval width.

(50) ℱ(θ) = ℱ_MSE_(θ) + λ_cov_ ⋅ ℱ_cov_(θ) + λ_width_ ⋅ ℱ_width_(θ) + 𝒫(θ)

Each component is defined as follows:

(1) Point estimation accuracy (MSE):

(51) $$F_{\mathrm{MSE}}(\theta )=\frac{1}{T}\sum_{t=1}^{T}({\hat{y}}_{t}(\theta )-y_{t}{)}^{2}$$

where T is the number of training samples, ${\hat{y}}_{t}(\theta )$ is the SOH point estimate of the tth sample under parameter set **θ**, and **y**_t_ is the true value.

(2) Coverage loss:

(52) $$F_{\mathrm{cov}}(\theta )=\max{\left(0,\alpha_{\mathrm{target}}-{\mathrm{ICP}}_{\mathrm{train}}(\theta )\right)}^{2}$$

where α_target_ is the target coverage probability, and ICP_train_(θ) is the actual interval coverage probability on the training set, defined as:

(53) $${\mathrm{ICP}}_{\mathrm{train}}(\theta )=\frac{1}{T}\sum_{t=1}^{T}I\{y_{t}\in [y_{L,t}(\theta ),y_{U,t}(\theta )]\}$$

The coverage loss is activated only when ICP_train_ < α_target_. The model is encouraged to achieve the target coverage probability.

(3) Width loss:

(54) $$F_{\mathrm{width}}(\Theta )={\left({\bar{W}}_{\mathrm{int}}(\Theta )-W_{\mathrm{target}}\right)}^{2}$$

where ${\bar{W}}_{\mathrm{int}}(\Theta )$ is the average interval width on the training set, defined as:

(55) $${\bar{W}}_{\mathrm{int}}(\Theta )=\frac{1}{T}\sum_{t=1}^{T}(y_{U,t}(\Theta )-y_{L,t}(\Theta ))$$

where W_target_ is the target width. The width loss encourages the model to produce compact estimation intervals.

(4)onstraint penalty term:

(56) $$P(\theta )=\sum_{k=1}^{L_{\mathrm{IBRB}}}\left[P_{k}^{\left(1\right)}(\theta )+P_{k}^{\left(2\right)}(\theta )\right]$$

(57) $$P_{k}^{\left(1\right)}(\theta )=\left\{\begin{array}{ll} 0, & \mathrm{if}\;\beta_{n,k}^{L}\leq \beta_{n,k}^{U},\forall n=1,2,\ldots ,N\\ 100, & \mathrm{otherwise} \end{array}\right.$$

(58) $$P_{k}^{\left(2\right)}(\theta )=\left\{\begin{array}{ll} 0, & \mathrm{if}\;\sum_{n=1}^{N}\beta_{n,k}^{L}\leq 1\leq \sum_{n=1}^{N}\beta_{n,k}^{U},\\ 50\cdot \left(\max(0,\sum_{n=1}^{N}\beta_{n,k}^{L}-1)+\max(0,1-\sum_{n=1}^{N}\beta_{n,k}^{U})\right), & \mathrm{otherwise} \end{array}\right.$$

(5) Loss weights:

The terms λ_cov_ = 2.0 and λ_width_ = 0.5 are the weight coefficients of the respective loss terms.

#### 3.4.4. P-CMA-ES Optimization Procedure

The search distribution is dynamically adjusted by the P-CMA-ES algorithm through the covariance matrix adaptation mechanism. Equality constraints are handled by incorporating a projection operator. The following steps are executed in each generation:

Step 1 (Parameter initialization): Algorithm parameters are set.

Parameter initialization: The population size λ and the selection population size μ are set as follows:

(59) $$\lambda =4+\left\lfloor 3\ln(D)\right\rfloor ,\mu =\left\lfloor \frac{\lambda }{2}\right\rfloor$$

where D = |Θ| is the dimension of the optimization variables.

The recombination weights ω_i_ are defined as:

(60) $$\omega_{i}=\frac{\ln(\mu +1/2)-\ln i}{\sum_{j=1}^{\mu }(\ln(\mu +1/2)-\ln j)}\sum_{i=1}^{\mu }\omega_{i}=1$$

The effective selection mass μ_eff_ is computed as:

(61) $$\mu_{\mathrm{eff}}={\left(\sum_{i=1}^{\mu }\omega_{i}^{2}\right)}^{-1}$$

The learning rate parameters are set as follows:

(62) $$c_{c}=\frac{4+\mu_{\mathrm{eff}}/D}{D+4+2\mu_{\mathrm{eff}}/D}$$

(63) $$c_{s}=\frac{\mu_{\mathrm{eff}}+2}{D+\mu_{\mathrm{eff}}+5}$$

(64) $$c_{1}=\frac{2}{{\left(D+1.3\right)}^{2}+\mu_{\mathrm{eff}}}$$

(65) $$c_{\mu }=\min\left(1-c_{1},\frac{2(\mu_{\mathrm{eff}}-2+1/\mu_{\mathrm{eff}})}{{\left(D+2\right)}^{2}+\mu_{\mathrm{eff}}}\right)$$

(66) $$d_{s}=1+2\max\left(0,\sqrt{\frac{\mu_{\mathrm{eff}}-1}{D+1}}-1\right)+c_{s}$$

The evolution paths are initialized as zero vectors: $p_{c}^{\left(0\right)}=0$ and $p_{s}^{\left(0\right)}=0$ The covariance matrix is initialized as the identity matrix: C^(0)^ = I. The global step size is initialized as σ^(0)^ = 0.5.

Step 2 (Sampling): A total of λ candidate solutions are generated from the multivariate normal distribution:

(67) $$\theta_{k}^{\left(g+1\right)}=m^{\left(g\right)}+\sigma^{\left(g\right)}B^{\left(g\right)}D^{\left(g\right)}z_{k},\;z_{k}\sim N(0,I),\;k=1,2,\ldots ,\lambda$$

where m^(g)^ is the distribution mean of the gth generation, σ^(g)^ is the global step size, C^(g)^ is the covariance matrix, and λ is the population size.

Step 3 (Boundary constraints and projection): The projection operator is applied to each candidate solution. Equality constraints and boundary constraints are ensured to be satisfied.

(68) $$\Theta_{k}^{\left(g+1\right)}\leftarrow \min\left(\max\left(\Theta_{k}^{\left(g+1\right)},1\right),u\right)$$

(69) $$\Theta_{k}^{\left(g+1\right)}\leftarrow P\left(\Theta_{k}^{\left(g+1\right)}|A_{\mathrm{eq}}\Theta =1\right)$$

where 1 and u are the lower and upper bound vectors of the variables, respectively. The projection operator is denoted as 𝒫(·).

Step 4 (Evaluation): The fitness value $F(\Theta_{k}^{\left(g+1\right)})$ is computed for each candidate solution.

Step 5 (Sorting and selection): All candidate solutions are sorted in ascending order of their fitness values. The μ individuals with the best fitness values are selected:

(70) $$\Theta_{1:\lambda }^{\left(g+1\right)},\Theta_{2:\lambda }^{\left(g+1\right)},\ldots ,\Theta_{\mu :\lambda }^{\left(g+1\right)}$$

where $\Theta_{i:\lambda }$ denotes the ith best individual in generation.

Step 6 (Mean update): The distribution mean is updated as follows:

(71) $$m^{\left(g+1\right)}=\sum_{i=1}^{\mu }\omega_{i}h_{s}^{\left(g+1\right)}=\left\{\begin{array}{ll} 1, & \mathrm{if}\;\frac{\|p_{s}^{\left(g+1\right)}{\|}^{2}}{1-{\left(1-c_{s}\right)}^{2(g+1)}}<2+\frac{4}{D+1}\\ 0, & \mathrm{otherwise} \end{array}\right.\theta_{i:\lambda }^{\left(g+1\right)}$$

Step 7 Step-size evolution path update:

(72) $$p_{s}^{\left(g+1\right)}=(1-c_{s})p_{s}^{\left(g\right)}+\sqrt{c_{s}(2-c_{s})\mu_{\mathrm{eff}}}\cdot {\left(C^{\left(g\right)}\right)}^{-1/2}\frac{m^{\left(g+1\right)}-m^{\left(g\right)}}{\sigma^{\left(g\right)}}$$

Step 8 The global step size is updated as follows:

(73) $$\sigma^{\left(g+1\right)}=\sigma^{\left(g\right)}\cdot \exp\left(\frac{c_{s}}{d_{s}}\left(\frac{\left\|p_{s}^{\left(g+1\right)}\right\|}{\chi_{D}}-1\right)\right)$$

Step 9 The evolution path and the covariance matrix are updated as follows:

(74) $$h_{s}^{\left(g+1\right)}=\left\{\begin{array}{ll} 1, & \mathrm{if}\;\frac{\|p_{s}^{\left(g+1\right)}{\|}^{2}}{1-{\left(1-c_{s}\right)}^{2(g+1)}}<2+\frac{4}{D+1}\\ 0, & \mathrm{otherwise} \end{array}\right.$$

(75) $$P_{c}^{\left(g+1\right)}=(1-c_{c})P_{c}^{\left(g\right)}+\sqrt{c_{c}(2-c_{c})\mu_{\mathrm{eff}}}\cdot \frac{m^{\left(g+1\right)}-m^{\left(g\right)}}{\sigma^{\left(g\right)}}$$

Step 10 Covariance matrix update:

(76) $$C^{\left(g+1\right)}=(1-c_{1}-c_{\mu })C^{\left(g\right)}+c_{1}P_{c}^{\left(g+1\right)}{\left(P_{c}^{\left(g+1\right)}\right)}^{⊤}+c_{\mu }\sum_{i=1}^{\mu }\omega_{i}y_{i:\lambda }^{\left(g+1\right)}{\left(y_{i:\lambda }^{\left(g+1\right)}\right)}^{⊤}$$

where $P_{c}$ is the evolution path, and $y_{i:\lambda }=(\theta_{i:\lambda }^{\left(g+1\right)}-m^{\left(g\right)})/\sigma^{\left(g\right)}$ is the evolution step of the ith elite individual. The parameters c_c_, c_1_, and c_μ_ are the learning rates for the evolution path, the rank-one update, and the rank- μ update, respectively. The effective selection mass is computed as μ_eff_.

Termination criterion: The iteration is terminated when the maximum number of generations G_max_ is reached. The optimal parameter set θ^*^ is then output.

**Figure 3:**
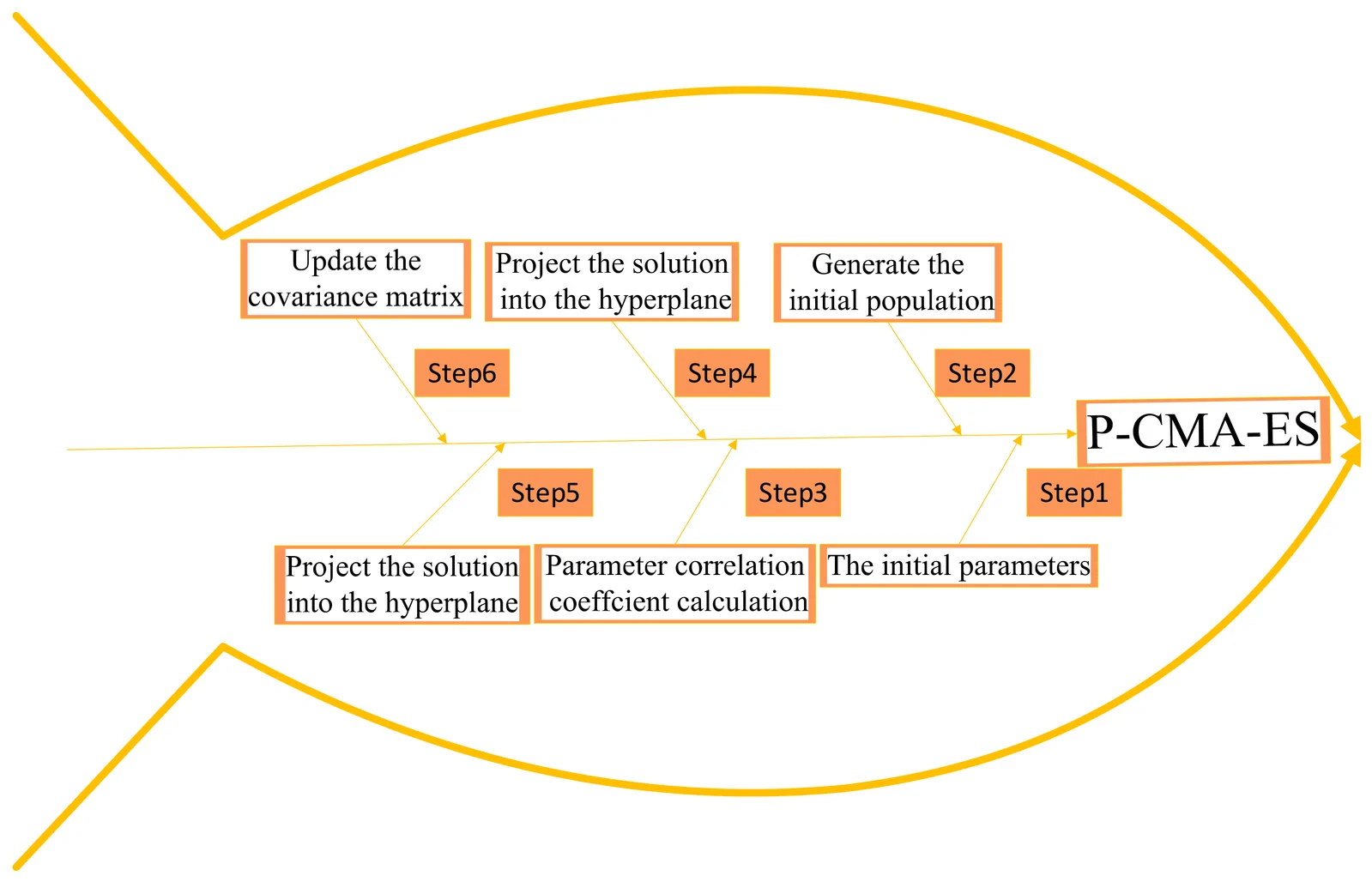
Flowchart of the P-CMA-ES optimization algorithm.

The above optimization process is iterated until convergence. The final output of the optimal parameter set θ^*^ is taken as the optimal parameter configuration of the IBRB-IER model. As shown in Figure 3.

The optimization process of the P-CMA-ES algorithm ensures the high accuracy of the IBRB-IER model, but it also introduces computational complexity. As demonstrated by recent work on fast parameter identification using the Padé approximation for simplified P2D models [34], model simplification techniques can significantly improve computational efficiency while maintaining acceptable accuracy.

This insight is highly valuable for our study. It indicates that exploring lightweighting strategies for the IBRB-IER model—such as rule base pruning or parameter sharing—while maintaining the current interval estimation accuracy will be a highly valuable direction to improve the model’s applicability in practical BMS.

### 3.5. Theoretical Properties and Analysis

#### 3.5.1. Sources of Uncertainty

The interval estimation generated by the IBRB-IER model inherently aggregates multiple sources of uncertainty. Instead of relying on a complex probabilistic decomposition framework, the sources of uncertainty are explicitly mapped to the model’s structural components:

(1) Parameter Uncertainty:

This is captured by the interval half-width δ_i,j_ within the belief degree intervals. δ_i,j_ represents the uncertainty in the rule’s output caused by limited expert knowledge and incomplete training data.

(2) Model Structure Uncertainty:

This is represented by the unassigned uncertainty mass m_D_ during the IER fusion process. m_D_ quantitatively reflects the ignorance or structural incompleteness when combining multiple rules.

(3) Measurement Noise and Operating Conditions:

The Gaussian membership function inherently smooths input measurement noise. When input features fluctuate due to noise or dynamic conditions, the matching degree α_i,j_ changes smoothly, preventing abrupt interval widening.

By explicitly representing these uncertainties as interval-valued belief degrees and propagating them through the IER algorithm, the IBRB-IER model provides a transparent and scientifically interpretable uncertainty quantification process.

#### 3.5.2. Computational Complexity Analysis

To evaluate the feasibility of deploying the IBRB-IER model on embedded BMS hardware, the theoretical time and space complexity are analyzed. The model demonstrates significant lightweight advantages in both aspects:

(1) Time Complexity:

During real-time operation, the model only performs Gaussian membership calculations and recursive interval ER combinations. The time complexity is O(L_act_ · N^2^). Since the number of activated rules is bounded and the evaluation grades are fixed, the online inference requires only a few basic arithmetic operations (addition and multiplication). This minimal computational load guarantees that the inference time is highly efficient, easily meeting the strict millisecond-level real-time requirements of BMS. The P-CMA-ES algorithm involves covariance matrix adaptation with a complexity of O(G · λ · D^2^). Although this phase is computationally intensive, it is executed entirely offline on a PC prior to deployment and does not consume the computational resources of the BMS microcontroller.

(2) Space Complexity:

The model’s memory footprint is strictly determined by the rule base parameters. The parameter set includes belief degrees, interval half-widths, reliabilities, and weights. For L_IBRB_ = 18 rules and N = 3 grades, the total number of parameters is 18 × (3 + 1 + 1 + 1) = 108 floating-point numbers. In a 32-bit format, this requires only 432 bytes of memory. Compared to the typical RAM capacity of ARM Cortex-M microcontrollers (e.g., 64 KB), the IBRB-IER model consumes less than 1% of the memory, demonstrating exceptional lightweight characteristics for embedded deployment.

#### 3.5.3. Physical Interpretability: Mapping Interval Widths to Aging Mechanisms

To enhance the physical interpretability of the IBRB-IER model, the interval half-width δ_i,j_ should not be viewed merely as a data-driven parameter, but as a mathematical manifestation of underlying electrochemical degradation mechanisms. The relationship between the interval width and specific aging modes is analyzed as follows:

(1) SEI Layer Growth (Narrow Interval): The formation and thickening of the Solid Electrolyte Interphase (SEI) is a gradual and relatively predictable process. The consumption of lithium ions during SEI growth leads to a smooth, monotonic capacity fade. Because this degradation mode is highly deterministic under normal operating conditions, the uncertainty in the extracted health features is low. Consequently, the optimization algorithm assigns a narrower interval width (δ_i,j_) to the corresponding rules.

(2) Lithium Plating (Wide Interval): Lithium plating typically occurs under harsh conditions, such as low temperatures or high charging rates. It is a highly non-linear and abrupt degradation mode. The onset of lithium plating causes rapid and irregular changes in internal resistance and voltage profiles. This sudden deviation induces significant epistemic and aleatoric uncertainty in the input data. To cover the true SOH value safely during these sudden degradations, the P-CMA-ES optimization automatically expands the uncertainty bounds, mapping to a wider interval width (δ_i,j_).

(3) Loss of Active Material (LAM) (Intermediate Interval): The loss of active material due to particle cracking or isolation is influenced by complex coupled factors (e.g., temperature, depth of discharge). Its degradation rate is moderate but highly dependent on dynamic operating conditions. Therefore, the uncertainty associated with this mechanism falls between that of SEI growth and lithium plating, mapping to an intermediate interval width.

#### 3.5.4. Robustness Mechanism Under Extreme Operating Conditions

In practical BMS applications, batteries inevitably face extreme conditions such as low temperatures (−20 °C), high State of Charge (90% SOC), and high-rate charging/discharging. Under these conditions, the electrochemical reaction kinetics change drastically, leading to severe data distribution shifts and feature degradation (e.g., significant voltage polarization and abnormal internal resistance variations).

While the current study validates the model under standard laboratory conditions, the IBRB-IER framework inherently possesses a robustness enhancement mechanism against such extreme scenarios:

(1) Extreme conditions cause sensor features to deviate from the training distribution. The Gaussian membership function in IBRB-IER naturally smooths the input features; when features degrade, the membership degrees smoothly transition rather than abruptly failing. This ensures the model does not produce erratic outputs even when input features fall into sparse regions.
(2) Under extreme conditions, the activated rules may conflict or exhibit high unreliability. Through the interval evidential reasoning (IER) fusion, the unassigned uncertainty mass *m_D_* will automatically increase. This dynamically widens the estimation interval, providing a conservative risk boundary that safely covers the true SOH, preventing false alarms in BMS.
(3) The belief degree intervals in the rule base embed expert knowledge regarding degradation limits. Even under distribution shifts, the physical boundaries encoded in the rules prevent the model from outputting physically impossible SOH values.

## 4. Experimental Validation

### 4.1. Experimental Definition

#### 4.1.1. Dataset Description

Lithium-ion batteries have been widely applied in aerospace and other fields. However, during their service life, internal electrochemical reactions continuously occur, leading to gradual capacity degradation. Once the degradation exceeds a certain threshold, spontaneous combustion or explosion may be triggered, potentially resulting in immeasurable losses.

In this paper, the NASA Lithium-ion Battery Aging Datasets are employed for experimental validation. These datasets were collected by the Prognostics Center of Excellence at the Ames Research Center of NASA using a customized battery prognostics test platform [35]. They are one of the most widely used public benchmark datasets in the field of battery health management (PHM), and have been extensively adopted by scholars worldwide for lithium battery SOH estimation, remaining useful life (RUL) prediction, and degradation modeling.

Four groups of lithium-ion battery datasets (numbered B0005, B0006, B0007, and B0018) are used for validation in this paper. The experimental data were obtained from cyclic aging tests of 18650 lithium-ion batteries, during which the cells were charged at a constant current of 1C to 4.2 V under a constant temperature of 25 °C, and then discharged at 1C to 2.75 V. This charge–discharge cycle was repeated until the battery capacity degraded to below 70% of the rated capacity.

#### 4.1.2. Feature Selection Criteria

The aging process of lithium-ion batteries involves multiple physicochemical mechanisms. To construct an effective SOH assessment model, the selection of antecedent attributes should satisfy the following principles: (1) a clear physical correlation with battery aging should be established; (2) online or offline measurement should be feasible in actual BMS; and (3) sufficient sensitivity to aging state variations should be provided. Based on these principles and the availability of experimental data, three key monitoring indicators are selected as model input features:

(1) Constant voltage charging time (unit: s). In the CC-CV charging mode, the constant voltage phase is initiated once the terminal voltage reaches 4.2 V, and the time taken for the charging current to decay from its maximum value to the cutoff current of 20 mA is measured.

(2) Constant current discharge time with equal voltage drop (unit: s). During constant current discharge, the time elapsed for the terminal voltage to drop from 4.0 V to 3.6 V is recorded.

(3) Charge transfer resistance (unit: Ω). This parameter is extracted through electrochemical impedance spectroscopy (EIS) tests and obtained by fitting the Randles equivalent circuit.

**Figure 4:**
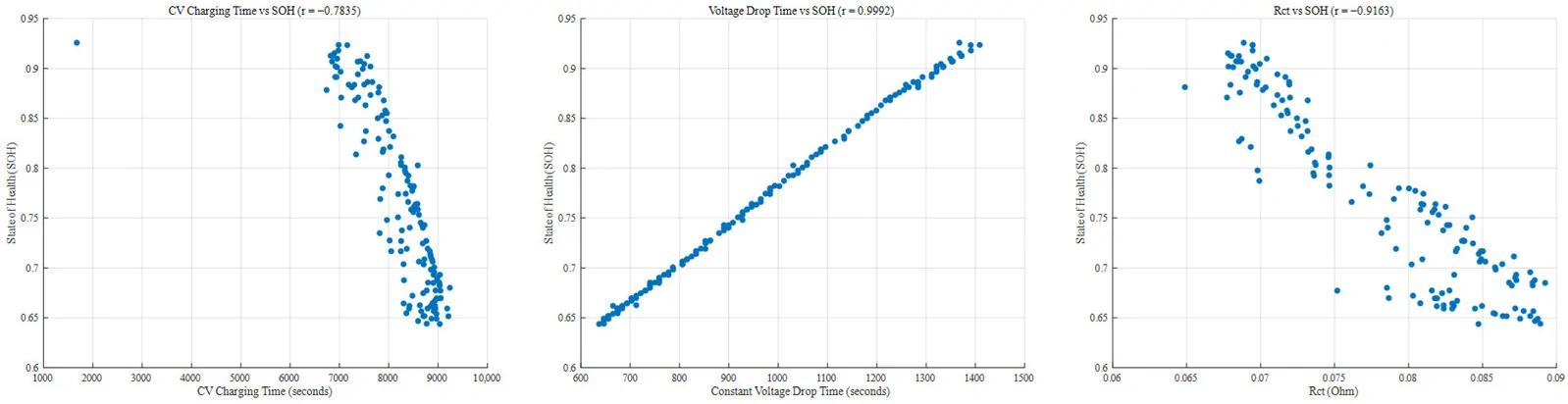
Feature extraction for battery B0005.

The above three features characterize battery aging states from three perspectives: the charging process, the discharging process, and internal electrochemical characteristics. Complementary information is provided by these features, as summarized in Table 2. The Pearson correlation coefficients between the three features and SOH are −0.7835, 0.9992, and −0.9163, respectively, indicating strong linear relationships in all cases. The effectiveness of the selected health factors is therefore validated, as illustrated in Figure 4.

Feature 2 exhibits an extremely high Pearson correlation coefficient (0.9992) with SOH, serving almost as a direct proxy for capacity degradation. While a simple deterministic mapping using this single feature could yield a competitive point estimation under ideal conditions, it falls short in practical BMS applications. Pure data mapping cannot quantify the reliability of the estimation. The primary contribution of the proposed IBRB-IER framework is not merely to utilize this strong feature, but to establish a reasoning mechanism that quantifies uncertainty. By incorporating interval belief degrees, the IBRB-IER model translates these features into quantifiable estimation intervals. This approach provides essential risk boundaries (with a 97.78% coverage probability) for battery management, which simple point-estimation mappings fail to achieve.

Meanwhile, the SOH label is defined as the ratio of the current available capacity to the rated capacity:

(77) $$\mathrm{SOH}=\frac{C_{\mathrm{current}}}{C_{\mathrm{rated}}}\times 100\%$$

where C_current_ is provided by the cumulative discharge capacity recorded in each discharge cycle, and C_rated_ = 2.0Ah is the rated capacity. A total of 168 cycles were completed for battery B0005. After data cleaning, 149 valid samples were obtained. These samples were divided into a training set (104 samples) and a test set (45 samples) at a ratio of 7:3.

**Figure 5:**
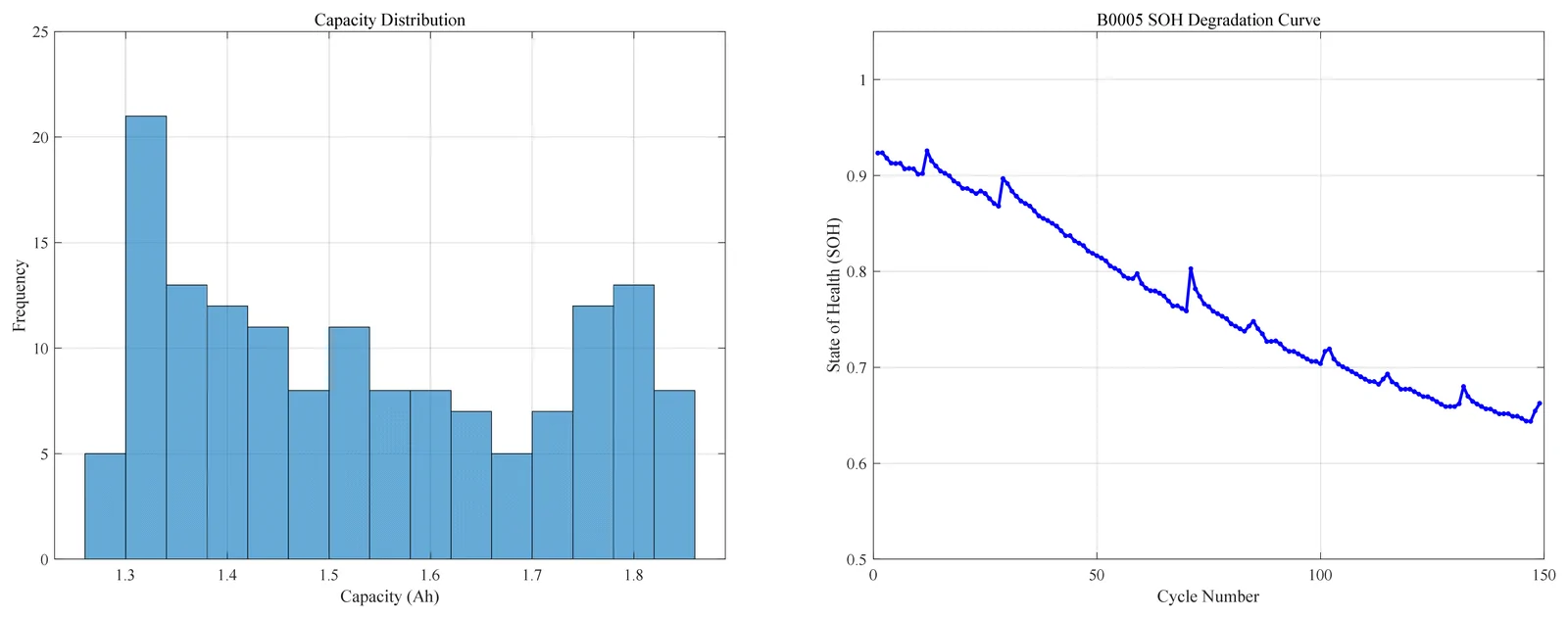
Capacity distribution and SOH distribution of battery B0005.

After feature extraction, 168 cycles were completed for battery B0005. To ensure the validity and reliability of the training data, the original data were cleaned, and invalid samples were removed based on the following criteria: (1) lack of forward charging data in the first cycle, preventing calculation of the constant voltage charging time; (2) missing voltage or current data causing feature extraction failure; (3) no valid impedance measurement data within the search range, or non-convergence of equivalent circuit fitting, making the charge transfer impedance unavailable; (4) feature values exceeding the physically reasonable range; and (5) SOH values outside the interval [0.1, 1.0]. After cleaning, 149 valid samples were obtained, accounting for 88.7% of the total cycles. To objectively evaluate the model’s generalization capability, the valid samples were randomly divided into a training set (104 samples) and a test set (45 samples) at a 7:3 ratio. The capacity and SOH distributions of battery B0005 are shown in Figure 5, where SOH values mainly range from 0.65 to 0.92, covering the complete degradation process from healthy to aged states, thereby providing sufficient sample support for model training and validation.

#### 4.1.3. Experimental Setup

In this study, the health state is characterized by the SOH value, which measures the degradation degree of current capacity relative to the rated capacity and thereby reflects the battery’s aging status. To construct the evaluation system of the interval BRB model, the health state is divided into three grades—Healthy, Sub-healthy, and Aged—as shown in Table 3. For model validation, valid samples of battery B0005 under the three health states were randomly sampled, yielding a total of 149 data samples. These were then divided into a training set (104 samples) and a test set (45 samples) at a 7:3 ratio, ensuring fairness and objectivity in model training and evaluation. The initial reference intervals of the attributes are presented in Table 4.

**Figure 6:**
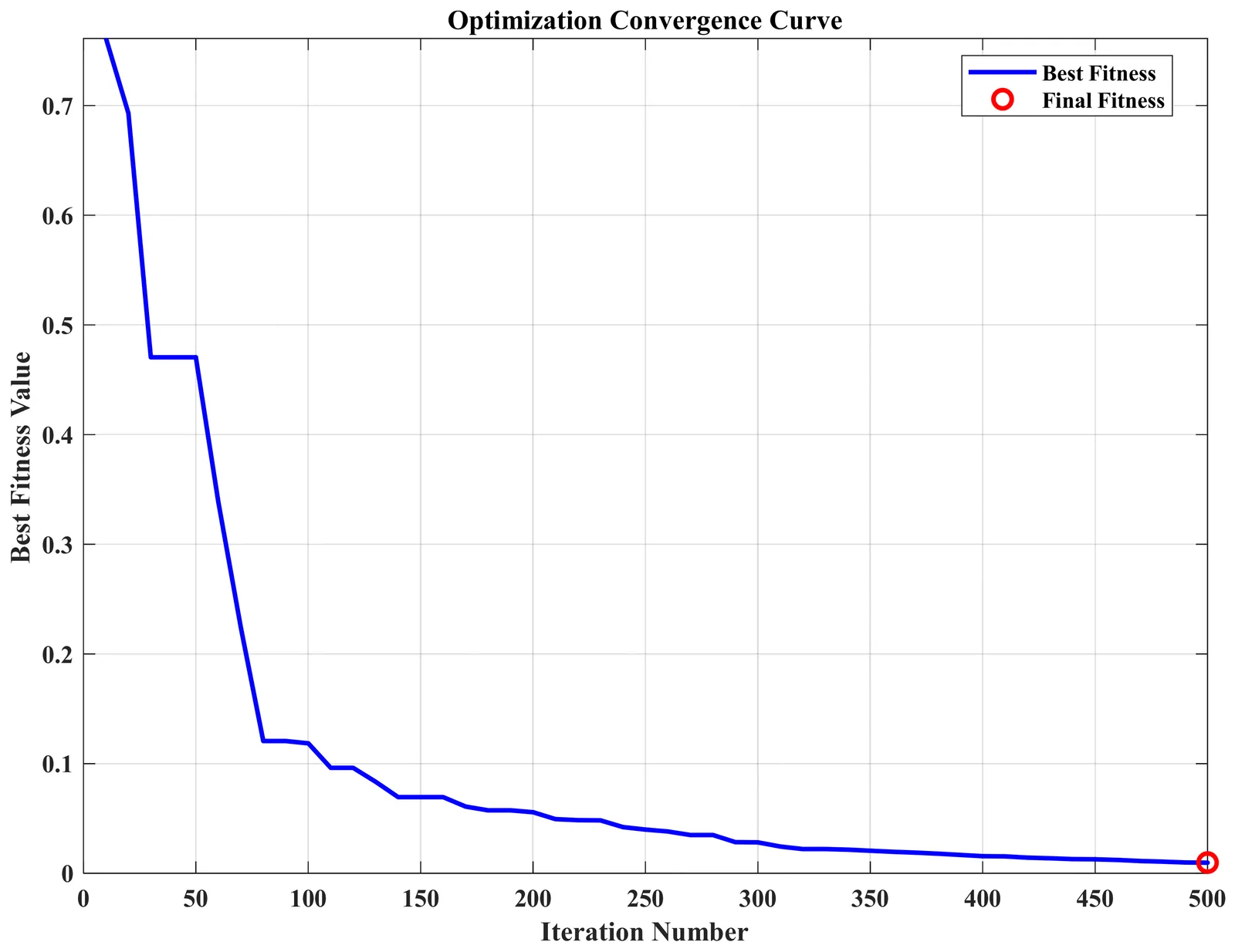
Convergence curve of the optimization algorithm.

The convergence curve of the optimization algorithm used in this paper is shown in Figure 6. As observed from the figure, the fitness value exhibits an overall trend of rapid decline followed by gradual stabilization as the number of iterations increases.

In the early stage (generations 0 to 50), the fitness value drops sharply from 0.75 to 0.12, indicating that strong global search capability is possessed by the algorithm in the initial stage. The region of the optimal solution can thus be quickly located, establishing a solid foundation for subsequent fine search. Between generations 50 and 150, the fitness value is further reduced to 0.07, but the decline rate becomes slower compared with the earlier stage, suggesting that the algorithm has transitioned from global exploration to local exploitation, with refined optimization performed around the current optimal solution. By generation 200, the fitness value has decreased to 0.05, after which the convergence speed becomes notably slower. At generation 300, the fitness value stabilizes at 0.01 and remains at this level throughout the subsequent iterations (generations 300 to 500), with the curve remaining flat and no significant fluctuations being observed. This indicates that the algorithm has converged to the vicinity of the global optimum and exhibits good stability.

**Figure 7:**
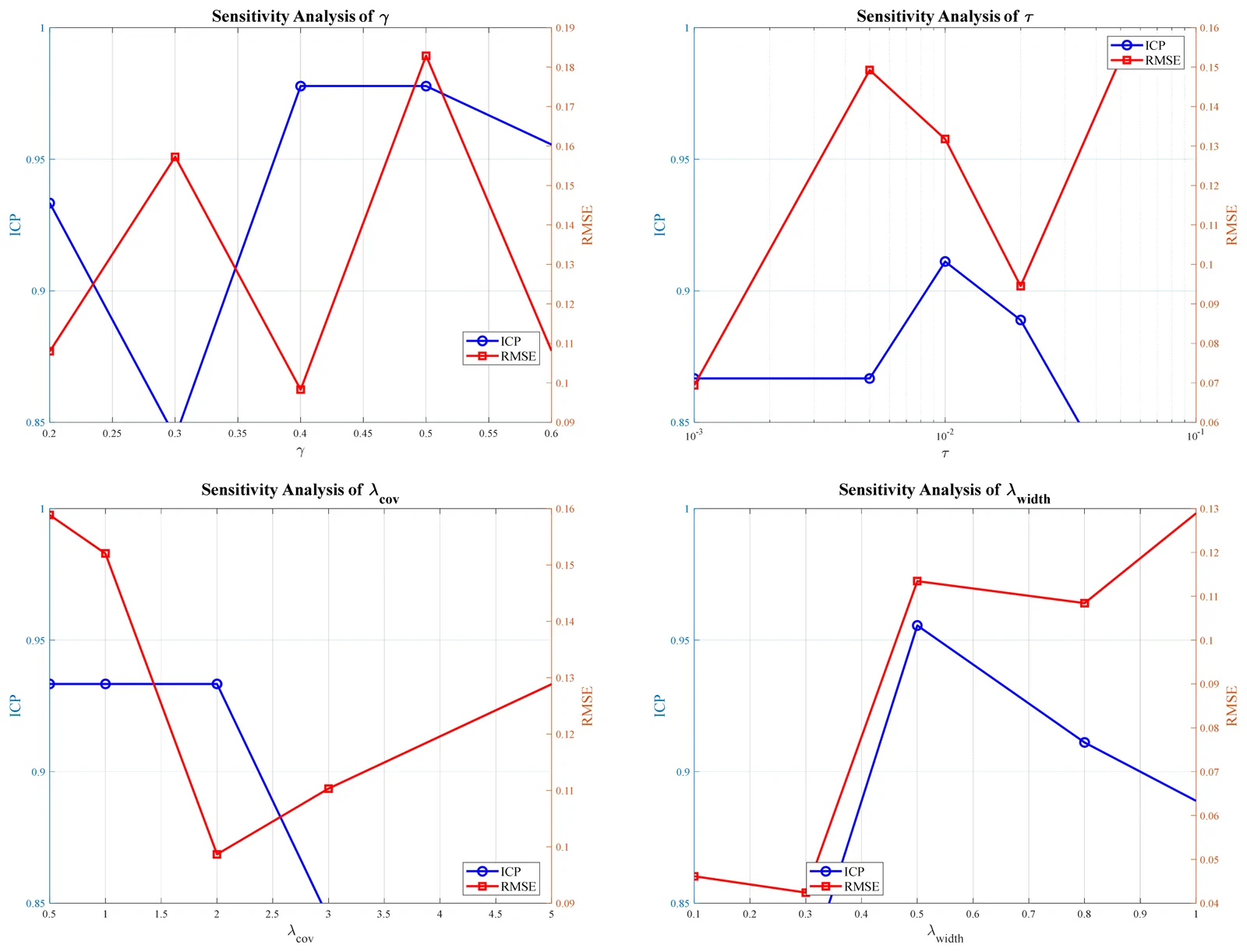
Sensitivity analysis of hyperparameters.

To validate the rationality of the configured hyperparameters, sensitivity analyses were conducted on the key hyperparameters, including the Gaussian width coefficient ϒ the activation threshold τ, the coverage loss weight λ_cov_, and the width loss weight λ_width_. The control variable method was adopted. Only one hyperparameter was changed at a time, while the others were kept at their default values. Each configuration was run independently. The results are shown in Table 5 and Figure 7.

The four key hyperparameters are all set within their respective performance-stable ranges. Experimental results show that the model performance remains stable when each hyperparameter is varied within a reasonable range, thereby confirming that the manual settings are supported by sufficient theoretical foundation and engineering rationality.

The key hyperparameters involved in this paper include the target coverage probability α_target, the target width W − target, the coverage loss weight λ_cov_, and the width loss weight λ_width_. Their setting rationale is as follows:

(1) Target coverage probability α_target = 0.90: This parameter represents the expected coverage probability for interval estimation. 90% is a commonly used confidence level in the field of interval estimation, providing sufficient reliability assurance for engineering decisions. Compared with 95% or higher confidence levels, the 90% target preserves reasonable margin for interval width optimization, avoiding overly conservative intervals that lose decision-making sensitivity.

(2) Target width W − target = 0.15: This parameter represents the expected average interval width on the training set. Since SOH is normalized to the range of [0, 1], an interval width of 0.15 corresponds to approximately 15% of the full SOH range. This value preserves reasonable tolerance for measurement errors and model uncertainty while providing sufficient sensitivity for battery maintenance decisions.

(3) Coverage loss weight λ_cov_ = 2.0 and width loss weight λ_width_ = 0.5: The ratio λ_cov_/λ_width_ = 4:1 reflects the optimization strategy of “prioritizing coverage satisfaction, then optimizing interval width.” When coverage falls below the target, the coverage loss term generates a larger gradient signal, driving the model to prioritize coverage improvement; once coverage meets the target, the width loss term dominates the optimization process, compressing interval width while maintaining coverage.

To validate the rationality of the above hyperparameter settings, sensitivity analyses were conducted on the four key hyperparameters, as shown in Figure 7 and Table 5. The experimental results demonstrate that the model maintains stable performance within reasonable ranges of each hyperparameter. Specifically:

For the Gaussian width coefficient γ, the model performance remains stable within [0.3, 0.5], and γ = 0.4 is located within this stable range, balancing the smoothness of membership functions and the discrimination between adjacent reference levels.

For the activation threshold τ, the model performance remains stable within [0.005, 0.02], and τ = 0.01 is located within this stable range, effectively filtering low-contribution rules while maintaining information completeness.

For the coverage loss weight λ_cov_, the ICP exceeds 0.95 when λ_cov_ ≥ 2.0. The value λ_cov_ = 2.0 is the minimum weight that satisfies the coverage requirement, avoiding over-penalization of interval width.

For the width loss weight λ_width_, the ICP reaches above 0.95 when λ_width_ ≥ 0.3. The value λ_width_ = 0.5 further optimizes interval compactness on this basis.

The above sensitivity analysis results confirm that the manually set hyperparameters are located within their respective performance-stable ranges, and the settings are supported by sufficient theoretical foundation and engineering rationality.

### 4.2. Evaluation Metrics

Eight evaluation metrics are adopted in this paper to comprehensively assess the model performance from two dimensions: interval quality and point estimation accuracy.

#### 4.2.1. Interval Quality Evaluation Metrics

(1) Interval Coverage Probability (ICP):

(78) $$\mathrm{ICP}=\frac{1}{T}\sum_{t=1}^{T}I\{y_{t}\in [L_{t},U_{t}]\}$$

where T is the number of test samples. The lower and upper bounds of the predicted interval for the tth sample are denoted as L_t_ and U_t_, respectively. The true value is denoted as y_t_.

(2) Average Interval Width (AIW):

(79) $$\mathrm{AIW}=\frac{1}{T}\sum_{t=1}^{T}\left(U_{t}-L_{t}\right)$$

where T is the number of test samples. The lower and upper bounds of the predicted interval for the tth sample are denoted as L_t_ and U_t_, respectively.

(3) Normalized Average Interval Width (N-AIW)

(80) $$N-\mathrm{AIW}=\frac{\mathrm{AIW}}{\max(y)-\min(y)}$$

The influence of the SOH magnitude is eliminated. Comparisons across different datasets are thereby facilitated.

(4) Interval Score (IS):

(81) IS = ICP − λ ⋅ N − AIW

The trade-off between coverage and width is comprehensively evaluated by the IS metric.

(5) Coverage Width-based Criterion (CWC):

(82) CWC = N-AIW + γ ⋅ max(0,(1 − α) − ICP)

Penalties are imposed by the CWC on models with insufficient coverage. Smaller values are preferred. When ICP ≥ α the following condition is satisfied: CWC = N-AIW

#### 4.2.2. Point Estimation Accuracy Evaluation Metrics

(6) Mean Squared Error (MSE):

(83) $$\mathrm{MSE}=\frac{1}{T}\sum_{t=1}^{T}{\left({\hat{y}}_{t}-y_{t}\right)}^{2}$$

where ${\hat{y}}_{t}$ is the SOH point estimate (interval midpoint) of the tth sample.

(7) Mean Absolute Error (MAE):

(84) $$\mathrm{MAE}=\frac{1}{T}\sum_{t=1}^{T}|{\hat{y}}_{t}-y_{t}|$$

(8) Root Mean Squared Error (RMSE):

(85) $$\mathrm{RMSE}=\sqrt{\mathrm{MSE}}$$

### 4.3. Experimental Results and Analysis

#### 4.3.1. Experimental Results

To validate the effectiveness of the proposed IBRB-IER model in lithium-ion battery SOH interval estimation, experiments were conducted on the test set samples, and the results are presented through visualization.

**Figure 8:**
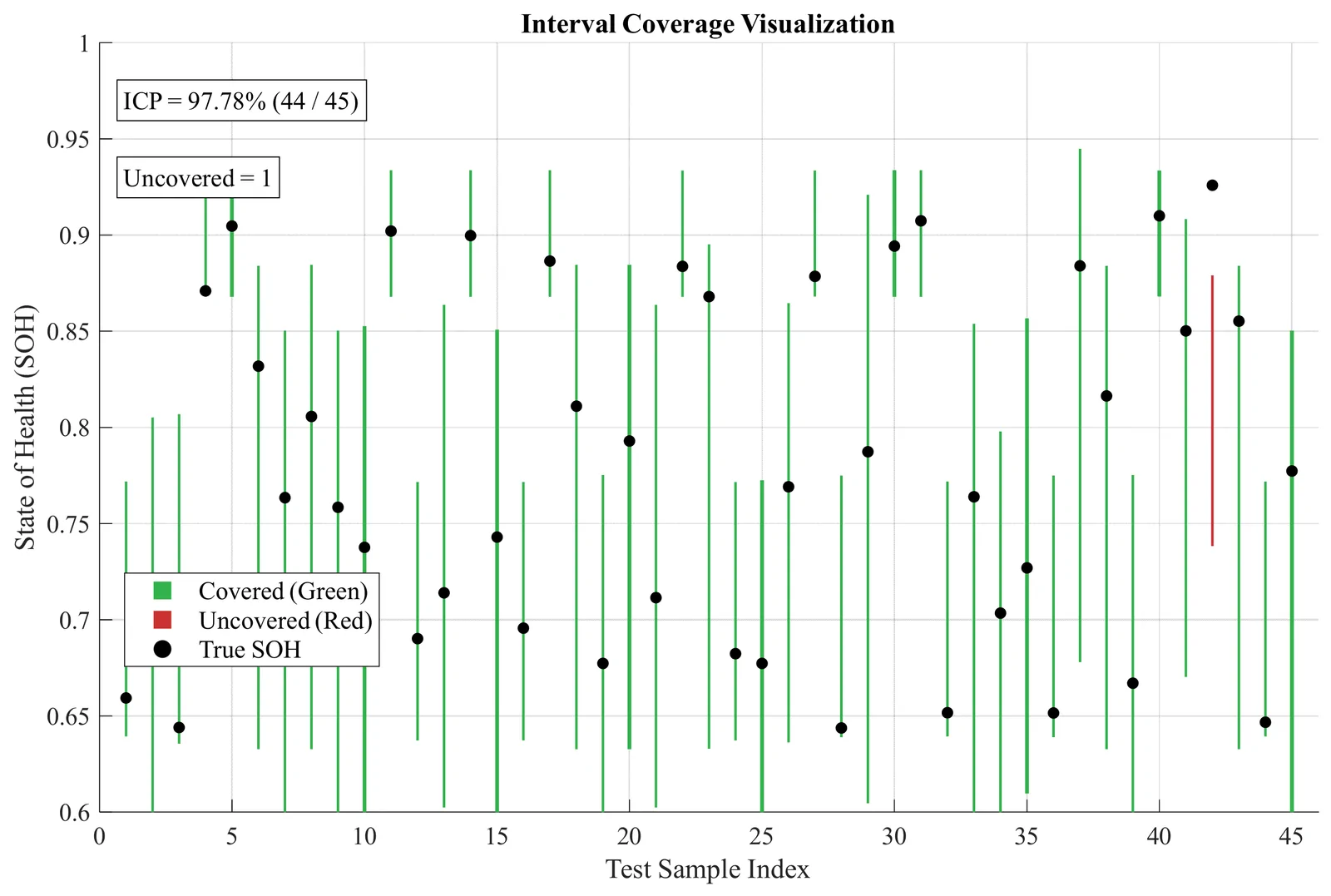
Interval coverage of battery B0005.

**Figure 9:**
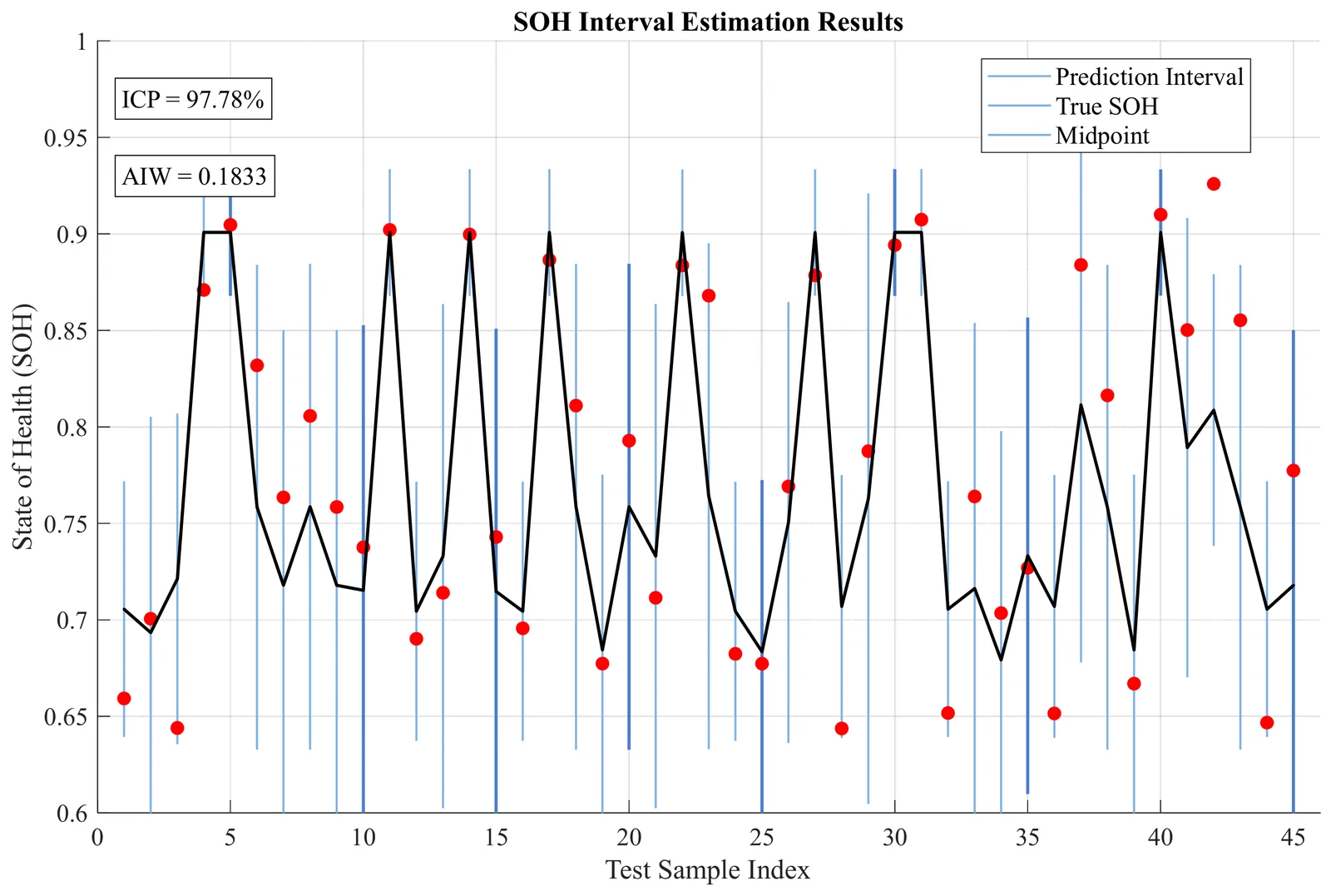
Interval estimation results of battery B0005. The black line represents the midpoint value, the blue lines represent the prediction interval, and the red dots represent the true State of Health (SOH).

As shown in Figure 8, effective coverage of the true SOH values is achieved by the proposed method on the vast majority of test samples. An ICP of 97.78% was obtained, significantly outperforming the preset target coverage of 90%. This demonstrates that the IBRB-IER model can provide highly reliable interval estimation results for SOH assessment. The corresponding interval estimation results are illustrated in Figure 9, where the blue vertical lines represent the prediction intervals, the red dots denote the true SOH values, and the blue dots indicate the interval midpoints.

The reliability of interval estimation depends not only on coverage probability but also on interval compactness. Overly wide intervals may easily cover the true values, yet the practical value of decision-making information is lost. Conversely, overly narrow intervals may frequently miss the true values, thereby reducing the credibility of the assessment. Therefore, an excellent interval estimation method must achieve a reasonable balance between coverage probability and interval width.

As shown in Figure 9, the prediction interval widths produced by the proposed method remain relatively stable across most samples, with no abnormally wide or narrow intervals observed. The AIW on the test set is 0.1833. Given that the true SOH values on the test set range from approximately 0.65 to 0.95, the N-AIW is around 0.61. This indicates that, while maintaining a high coverage probability of 97.78%, relatively compact interval widths are achieved by the model. The trade-off between interval coverage reliability and compactness is therefore well balanced, without sacrificing practical utility for high coverage.

The AIW achieved by the IBRB-IER model on the test set is 0.1833. To evaluate its practical applicability in battery SOH management, the physical meaning of this width must be considered. In real-world engineering, a typical battery is considered to reach its End of Life when its SOH drops to 0.70 or 0.80. The obtained AIW of 0.1833 means that for a predicted SOH of, for example, 0.75, the true SOH is highly likely to fall within [0.658, 0.841]. From a decision-making perspective, this interval width is highly applicable for several reasons:

The interval provides a clear risk boundary. Even in the worst-case scenario (the lower bound), if the SOH is 0.658, the battery is still above the critical failure threshold (0.60), meaning it can safely serve the current operational cycle without immediate risk of sudden failure. Conversely, a much wider interval (e.g., AIW > 0.30) would span the EOL threshold, rendering the assessment useless for decision-making (e.g., predicting SOH ∈ [0.55, 0.95] provides no actionable information). The IBRB-IER model successfully maintains a balance, providing a compact enough interval that guarantees high decision sensitivity while retaining 97.78% reliability.

It should be noted that randomly partitioning the dataset from a single battery (e.g., B0005) means the training and testing sets share similar degradation stages and local characteristics. Due to the temporal continuity of battery aging, adjacent cycles exhibit high autocorrelation. This partitioning strategy primarily tests the model’s local interpolation capability within a known degradation trajectory. It effectively demonstrates the model’s ability to capture the underlying nonlinear relationship between health features and SOH. However, it may overestimate the model’s extrapolation capability to unseen batteries with different initial states or varying aging rates. To compensate for this and evaluate the model’s cross-battery generalization, independent experiments on four distinct batteries (B0005, B0006, B0007, and B0018) were further conducted in Section 4.3.2, which confirmed the robustness of the IBRB-IER model.

#### 4.3.2. Multi-Battery Experimental Validation

To evaluate the model’s adaptability and cross-battery performance, four distinct cells (B0005, B0006, B0007, and B0018) from the NASA dataset were selected for independent experiments. It should be noted that the training and testing data were partitioned independently within each cell. While this setup evaluates the within-battery validation (fitting capability to a specific degradation trajectory), comparing the performance across all four independent batteries rigorously demonstrates the model’s overall adaptability to varying battery characteristics. The raw data of each battery were cleaned and then randomly divided into training and test sets at a 7:3 ratio, with the dataset sizes summarized in Table 6. All experiments were conducted in 20 independent runs, and the average values of the evaluation metrics were taken as the final results, thereby eliminating the influence of random partitioning and parameter initialization.

The average interval evaluation metrics and their standard deviations of the IBRB-IER model across 20 experiments on the four batteries are presented in Table 7. These metrics include ICP, AIW, N-AIW, IS, and CWC.

In terms of interval coverage probability, the IBRB-IER model achieved ICP values above 90% on all four batteries, with values of 93.78%, 90.31%, 91.01%, and 90.54%, respectively. As shown in Table 7, the standard deviations remain small (ranging from 0.029 to 0.053), confirming that the model maintains stable and reliable interval estimation performance.

In terms of average interval width, the AIW values of the four batteries fall within the range of 0.17 to 0.20. The standard deviations remain small (ranging from 0.016 to 0.035), showing good consistency.

The IS integrates both coverage probability and interval width. The highest IS (0.6494) was achieved by B0006, while the lowest IS (0.4732) was obtained by B0007. This discrepancy is mainly attributed to the larger normalized interval width (N-AIW = 0.8738) of B0007, which significantly reduces the IS. The CWC, which also reflects the comprehensive performance of interval estimation, is highly correlated with N-AIW, further demonstrating the important role of interval width in comprehensive evaluation.

The point estimation accuracy metrics and their standard deviations of the IBRB-IER model on the four batteries are shown in Table 8, including MSE, MAE, and RMSE.

From Table 8, it can be observed that the IBRB-IER model achieves good point estimation accuracy on all four batteries. The RMSE values range between 0.051 and 0.070, and the MAE values range between 0.040 and 0.054.

Based on the experimental results on the four batteries, the IBRB-IER model demonstrates good consistency in both interval evaluation and point estimation. The ICP values of the four batteries remain stable within the range of 90% to 94%, indicating stable coverage capability across different battery aging data without significant performance fluctuations caused by individual battery differences. The AIW values are also stable, ranging between 0.17 and 0.20, showing that the model can effectively adapt to different data distribution characteristics while maintaining good generalization capability in interval compactness. Similarly, the RMSE values are stable between 0.051 and 0.070, demonstrating that reasonable point estimation results are provided by the model, with no complete failure observed on any specific cell.

To rigorously evaluate the model’s generalization capability to completely unseen batteries, a Leave-One-Battery-Out (LOBO) cross-validation experiment was conducted. In each iteration, three batteries were used as the training set, and the remaining unseen battery was used as the test set. The experimental results (mean ± standard deviation of multiple runs) are summarized in Table 9.

As shown in Table 9, when predicting completely unseen batteries with different physical initial states and degradation trajectories, the IBRB-IER model maintains highly consistent and robust performance. Specifically, the Interval Coverage Probability (ICP) remains above 92.1% across all four independent test sets, demonstrating that the uncertainty quantification capability of the model successfully transfers to unseen data. The Average Interval Width (AIW) is stably controlled within the narrow range of 0.208 to 0.242, indicating that the model does not generate overly conservative intervals when facing unseen batteries. Furthermore, the RMSE and MAE values are consistently low, ranging from 0.059 to 0.065 and 0.043 to 0.053, respectively. This cross-battery validation convincingly proves that the rule-based inference mechanism of IBRB-IER captures the generalized degradation characteristics of lithium batteries rather than overfitting a specific cell.

The above results confirm the effectiveness and generalization capability of the IBRB-IER model in lithium battery SOH interval assessment. To further rigorously evaluate its generalization under different data partitioning conditions, four lithium-ion batteries were selected, and systematic comparative experiments were conducted under four training set proportions: 90%, 80%, 70%, and 50%. The evaluation metrics, including ICP, AIW, MSE, MAE, and RMSE, are reported in Table 10, Table 11, Table 12 and Table 13.

Based on the statistical results of 16 sets of experiments across four batteries and four training proportions, the following conclusions can be drawn:

In all experiments on the four batteries, the optimal values on the vast majority of evaluation metrics were obtained with a training proportion of 70%. At this proportion, the highest ICP, the smallest AIW, and the lowest error metrics were achieved, indicating that a 70% training proportion provides the best balance between model learning sufficiency and generalization capability. High prediction accuracy and interval coverage quality were maintained across different training proportions for all batteries: ICP remained above 0.89 under all experimental conditions, and RMSE was generally kept within 0.085. This demonstrates good adaptability of the proposed method to battery cells and data scales, without obvious overfitting or underfitting phenomena. Even under the extreme condition of only 50% training data, prediction performance declined only moderately, yet remained within an acceptable range, with interval coverage rates all exceeding 0.893. All results are reported as averages over 20 repeated runs, and the stability of the evaluation metrics across experiments is reflected by the mean values, confirming that stable and reliable predictions can still be produced under random initialization or data partitioning fluctuations.

### 4.4. Comparative Experiments

#### 4.4.1. Comparison with Traditional BRB

To validate the effectiveness of the proposed IBRB-IER interval assessment method, the BRB-ER and IBRB-ER models were selected as comparison methods. The same training and test set partitions and optimization algorithm were adopted in all comparative experiments, thereby ensuring fairness of the comparison. The experimental results are presented in Table 14.

As can be observed from Table 14, the proposed IBRB-IER method achieved the optimal performance on three evaluation metrics.

Compared with BRB-ER, the proposed method achieved reductions of 81.6%, 57.7%, and 60.9% in MSE, RMSE, and MAE, respectively. This significant improvement is mainly attributed to the effective characterization of rule uncertainty by interval belief degrees, which enables better robustness to noisy data and boundary samples.

Compared with IBRB-ER—which also adopts interval belief degrees but uses traditional ER fusion—the proposed method achieved reductions of 57.0%, 34.7%, and 26.8% in MSE, RMSE, and MAE, respectively. The superiority of the IER algorithm in handling interval-type belief information is thereby validated. Traditional ER often employs approximation or midpoint substitution strategies when processing interval inputs, leading to information loss. In contrast, IER preserves complete interval information and maintains strict propagation of lower and upper bounds throughout the fusion process, thereby yielding more accurate fusion results.

#### 4.4.2. Comparison with Data-Driven Methods

To comprehensively validate the effectiveness of the proposed IBRB-IER interval assessment method, five typical methods were selected as comparison benchmarks: Back Propagation Neural Network (BPNN), K-Nearest Neighbors (KNN), Support Vector Regression (SVR), Extreme Learning Machine (ELM), Long Short-Term Memory (LSTM), and a deep neural network representing advanced representation learning (Transformer-based). The same training/test set partitions and evaluation protocol were adopted across all comparative experiments, thereby ensuring fairness and reproducibility. The experimental results are presented in Table 15.

The IBRB-IER model significantly outperformed all other comparison models in terms of RMSE and MAE, demonstrating superior point estimation accuracy. Specifically, in terms of RMSE, the proposed method achieved 0.038012, outperforming the second-best Transformer-based deep network (0.040598) by approximately 6.3%. Compared to other advanced deep learning models like LSTM (0.043677) and traditional machine learning models like KNN (0.056662), the reductions were 13.0% and 32.9%, respectively. Even greater improvements were observed compared to ELM, SVR, and BPNN. As RMSE is more sensitive to large errors, this substantial reduction strongly demonstrates the model’s robustness against extreme prediction deviations.

It is worth noting that while the Transformer-based deep network achieved a slightly lower MSE (0.001648), it failed to surpass IBRB-IER in RMSE and MAE. This indicates that deep learning models tend to overfit certain samples in small-sample scenarios, resulting in higher extreme errors. In contrast, the rule-based IBRB-IER framework maintains stable and balanced estimation performance.

More importantly, the IBRB-IER model is the only method capable of providing uncertainty quantification. It achieves an ICP above 90% on the test set with an average interval width controlled between 0.17 and 0.20. In contrast, all other comparison methods (including LSTM and Transformer) can only produce single point estimates, leaving the uncertainty of the assessment unquantified and limiting their applicability in safety-critical BMS applications.

### 4.5. Experimental Summary

To comprehensively evaluate the effectiveness of the proposed IBRB-IER model in lithium-ion battery SOH interval assessment, three sets of experiments were conducted. The first set verified the interval estimation performance on the B0005 battery dataset, achieving an ICP of 97.78% with an AIW of 0.1833. The second set extended the model to four batteries (B0005, B0006, B0007, and B0018) and validated its generalization capability across different aging trajectories. The third set compared the IBRB-IER model with traditional BRB-ER, IBRB-ER, and several mainstream data-driven methods, comprehensively evaluating both point estimation accuracy and interval estimation capability. The results confirm that the IER algorithm has notable advantages in preserving and propagating interval information. The IBRB-IER model achieves leading point estimation accuracy while also providing estimation intervals for SOH estimates, demonstrating excellent performance in interval coverage, generalization, and estimation accuracy. The effectiveness of combining interval belief rule bases with IER is thereby validated.

## 5. Conclusions

In this study, an interval SOH assessment method based on IBRB-IER was proposed to address two key limitations: the difficulty of quantifying uncertainty in SOH assessment and the inability of traditional BRB methods to provide more than point estimates. The IBRB serves as the inference core, integrated with IER to preserve interval information and produce SOH estimation intervals as outputs. Compactness of the rule base is achieved through interval-based design, while parallel activation of multiple rules is realized by combining Gaussian membership functions with threshold screening. A multi-objective fitness function is designed, and global optimization is performed using the P-CMA-ES algorithm. The effectiveness and generalization capability of the model are validated through experiments on the NASA dataset.

Future research can be conducted in the following directions. The SOH interval assessment results can be combined with RUL prediction to construct a complete framework from state assessment to life prediction. Using the upper and lower bounds of interval estimates, RUL prediction intervals can be further derived, providing more forward-looking risk evidence for battery maintenance and replacement decisions. In addition, further validation on multiple datasets in industrial scenarios should be carried out, along with systematic analyses of hyperparameter sensitivity, noise robustness, and algorithm stability. The generalization capability and engineering applicability of the method can thus be examined under a wider range of conditions, promoting its deployment and application in actual battery management systems.

## Figures and Tables

**Table 1:** Comparison between the proposed IBRB-IER and existing IBRB variants.

Method	Uncertainty Representation	Interval Inference Mechanism	Optimization Objective Function	Interval Output
IBRB-C	Interval belief degree	Traditional ER	Accuracy & Interpretability	✗
BRB-I	Point belief degree	Traditional ER	Accuracy & Interpretability	✗
IBRB-r	Interval belief degree	Traditional ER	Accuracy	✗
IBRB-IER	Interval belief degree	Interval ER	Accuracy + Coverage loss + Width loss	✓

Note: “✗” indicates “No” (or Absence), and “√” indicates “Yes” (or Presence).

**Table 2:** Dataset feature description.

Feature	Symbol	Description	Range
Feature 1	X1	Capacity degradation feature	[1674, 9239]
Feature 2	X2	Internal resistance variation feature	[636, 1408]
Feature 3	X3	Voltage characteristic factor	[0.0649, 1.8518]
Output	Y	SOH	[0.6437, 0.9259]

**Table 3:** Three health states.

Health States	H	SH	A
Reference Value	1	0.5	0

**Table 4:** Initial reference intervals of attributes.

Attribute	Referential Intervals
Constant-Voltage Charging Time	[1670, 7000, 7500, 8000, 8500, 9500]
Equal Voltage Drop Discharge Time	[630, 750, 850, 950, 1100, 1410]
Charge Transfer Resistance	[0.064, 0.070, 0.075, 0.080, 0.085, 0.090]

**Table 5:** Hyperparameter settings of the IBRB-IER model.

Hyperparameter	Symbol	Value
Gaussian width coefficient	ϒ	0.4
Activation threshold	τ	0.01
Coverage loss weight	λ_cov_	2.0
Width loss weight	λ_width_	0.5

**Table 6:** Data sizes of each battery.

Battery	Total Samples	Training Samples	Testing Samples
B0005	149	104	45
B0006	108	76	32
B0007	149	104	45
B0018	125	88	37

**Table 7:** Interval evaluation performance of the IBRB-IER model on four batteries (mean ± standard deviation over 20 experiments).

	ICP	AIW	N_AIW	IS	CWC
B0005	0.937778 ± 0.0293	0.172351 ± 0.0168	0.628109 ± 0.0615	0.623723 ± 0.0428	0.628109 ± 0.0615
B0006	0.903125 ± 0.0404	0.195597 ± 0.0353	0.507506 ± 0.0967	0.649372 ± 0.0549	0.507506 ± 0.0967
B0007	0.910053 ± 0.0533	0.202793 ± 0.0306	0.873768 ± 0.1387	0.473169 ± 0.0569	0.873768 ± 0.1387
B0018	0.905405 ± 0.0377	0.191227 ± 0.0259	0.777136 ± 0.0983	0.516837 ± 0.0580	0.777136 ± 0.0983

**Table 8:** Point estimation accuracy of the IBRB-IER model on four batteries (mean ± standard deviation over 20 experiments).

	MSE	MAE	RMSE
B0005	0.001489 ± 0.0005	0.030941 ± 0.0053	0.038012 ± 0.0053
B0006	0.00544 ± 0.0038	0.053515 ± 0.0196	0.069724 ± 0.0248
B0007	0.00361 ± 0.0018	0.046416 ± 0.0122	0.058267 ± 0.0150
B0018	0.0028 ± 0.0014	0.040268 ± 0.0100	0.051323 ± 0.0132

**Table 9:** Leave-One-Battery-Out cross-validation results (mean ± standard deviation).

TEST	ICP	AIW	MSE	MAE	RMSE
B0005	0.933557 ± 0.0354	0.242189 ± 0.0236	0.004634 ± 0.0030	0.053573 ± 0.0166	0.065405 ± 0.0198
B0006	0.931477 ± 0.0364	0.213952 ± 0.0256	0.003668 ± 0.0008	0.049695 ± 0.0057	0.060190 ± 0.0073
B0007	0.927133 ± 0.0402	0.213653 ± 0.0256	0.004099 ± 0.0013	0.050797 ± 0.0068	0.063297 ± 0.0010
B0008	0.921000 ± 0.0371	0.208226 ± 0.0388	0.004106 ± 0.0035	0.043866 ± 0.0152	0.059903 ± 0.0243

**Table 10:** Evaluation metrics of battery B0005 under different training proportions (mean ± standard deviation over 20 experiments).

B0005	ICP	AIW	MSE	MAE	RMSE
90%	0.916667 ± 0.0296	0.189587 ± 0.0354	0.002558 ± 0.0020	0.039072 ± 0.0153	0.047536 ± 0.0177
80%	0.920000 ± 0.0381	0.205325 ± 0.0541	0.004137 ± 0.0036	0.047571 ± 0.0242	0.058122 ± 0.0283
70%	0.937778 ± 0.0293	0.172351 ± 0.0168	0.001489 ± 0.0005	0.030941 ± 0.0053	0.038012 ± 0.0053
50%	0.918919 ± 0.0333	0.203267 ± 0.0498	0.00487 ± 0.0046	0.049326 ± 0.0242	0.063205 ± 0.0303

**Table 11:** Evaluation metrics of battery B0006 under different training proportions (mean ± standard deviation over 20 experiments).

B0006	ICP	AIW	MSE	MAE	RMSE
90%	0.893048 ± 0.0357	0.219255 ± 0.0358	0.00621 ± 0.0644	0.061215 ± 0.0161	0.076617 ± 0.0190
80%	0.896970 ± 0.0437	0.228421 ± 0.0468	0.00572 ± 0.0031	0.058785 ± 0.0158	0.073584 ± 0.0182
70%	0.903125 ± 0.0404	0.195597 ± 0.0353	0.00544 ± 0.0038	0.053515 ± 0.0196	0.069724 ± 0.0248
50%	0.893519 ± 0.0446	0.237665 ± 0.0352	0.00778 ± 0.0038	0.066508 ± 0.0178	0.085486 ± 0.0224

**Table 12:** Evaluation metrics of battery B0007 under different training proportions (mean ± standard deviation over 20 experiments).

B0007	ICP	AIW	MSE	MAE	RMSE
90%	0.893333 ± 0.0454	0.216349 ± 0.0380	0.00438 ± 0.0029	0.051465 ± 0.0178	0.063535 ± 0.0190
80%	0.938333 ± 0.0450	0.212213 ± 0.0366	0.00487 ± 0.0033	0.050460 ± 0.0171	0.065882 ± 0.0237
70%	0.910053 ± 0.0533	0.202793 ± 0.0306	0.00361 ± 0.0018	0.046416 ± 0.0122	0.058267 ± 0.0150
50%	0.921622 ± 0.0507	0.219893 ± 0.0430	0.00490 ± 0.0025	0.0537936 ± 0.0133	0.067951 ± 0.0174

**Table 13:** Evaluation metrics of battery B0018 under different training proportions (mean ± standard deviation over 20 experiments).

B0018	ICP	AIW	MSE	MAE	RMSE
90%	0.894737 ± 0.0367	0.220261 ± 0.0342	0.00404 ± 0.0022	0.050302 ± 0.0152	0.061133 ± 0.0179
80%	0.902000 ± 0.0458	0.226224 ± 0.0382	0.00327 ± 0.0019	0.044045 ± 0.0150	0.054881 ± 0.0164
70%	0.905405 ± 0.0377	0.191227 ± 0.0259	0.0028 ± 0.0014	0.040268 ± 0.0100	0.051323 ± 0.0132
50%	0.898094 ± 0.0473	0.218015 ± 0.0473	0.00377 ± 0.0027	0.045590 ± 0.0163	0.057808 ± 0.0212

**Table 14:** Comparison with different IBRB models.

	IBRB-IER	IBRB-ER	BRB-ER
MSE	0.001489	0.003462	0.008101
RMSE	0.038012	0.058244	0.089872
MAE	0.030941	0.042251	0.079077

**Table 15:** Comparison with different data-driven methods.

	IBRB-IER	BPNN	KNN	SVM	ELM	LSTM	Transformer
MSE	0.001489	0.007397	0.004008	0.005058	0.004875	0.002231	0.001648
RMSE	0.038012	0.082984	0.056662	0.071082	0.069076	0.043677	0.040598
MAE	0.030941	0.069661	0.063142	0.064225	0.052975	0.036486	0.035324

## Data Availability

The data used in this study are publicly available from the NASA Prognostics Center of Excellence (PCoE) lithium-ion battery aging dataset. These data were derived from publicly available sources and are not subject to privacy restrictions.
